# Open-source hardware design and construction of a modular robotics kit for STEM education

**DOI:** 10.1016/j.ohx.2026.e00819

**Published:** 2026-08-22

**Authors:** Luis Calle-Arévalo, Juan Francisco Gonzales, John Alonso Ortega Naula, Diego Gabriel Zamora Lituma

**Affiliations:** Grupo de Investigación Aprender a Aprender (GIAA), Universidad Politécnica Salesiana, Calle Vieja 12-30 y Elia Liut, Cuenca, Azuay, 010102, Ecuador

**Keywords:** Open Source Hardware (OSHW), Educational robotics, Arduino, 3D printing, Embedded systems, STEM education

## Abstract

Commercial educational kits for technology instruction often operate as closed ”black boxes,” presenting high import costs that limit their adoption in developing regions such as South America. This work presents an open-source, 5-module modular experimentation kit designed for STEM education in embedded systems and automation. Centered around an Arduino UNO core, the kit features progressive difficulty levels across five distinct modules: an environmental smart pot, an automated parking lot, an electromechanical drawbridge, a timed food dispenser, and a high-density home automation hub. The physical infrastructure combines low-cost FDM 3D printing with single-layer custom PCBs developed in Altium Designer. The total hardware cost is approximately $548.00 USD, optimized for localized manufacturing. Field validation with 40 s ondary school students in Cuenca, Ecuador, over a station-based collaborative rotation workshop demonstrated 100% hardware reliability under continuous operation. Quantitative post-workshop evaluations using a 5-point Likert scale indicated notable student engagement, curiosity, and positive motivation towards automation engineering concepts across all design modules. Complete parametric CAD files, manufacturing-ready Gerbers, firmware, and user manuals are fully open-access to promote global educational replication.

## Graphical abstract

**Figure:**
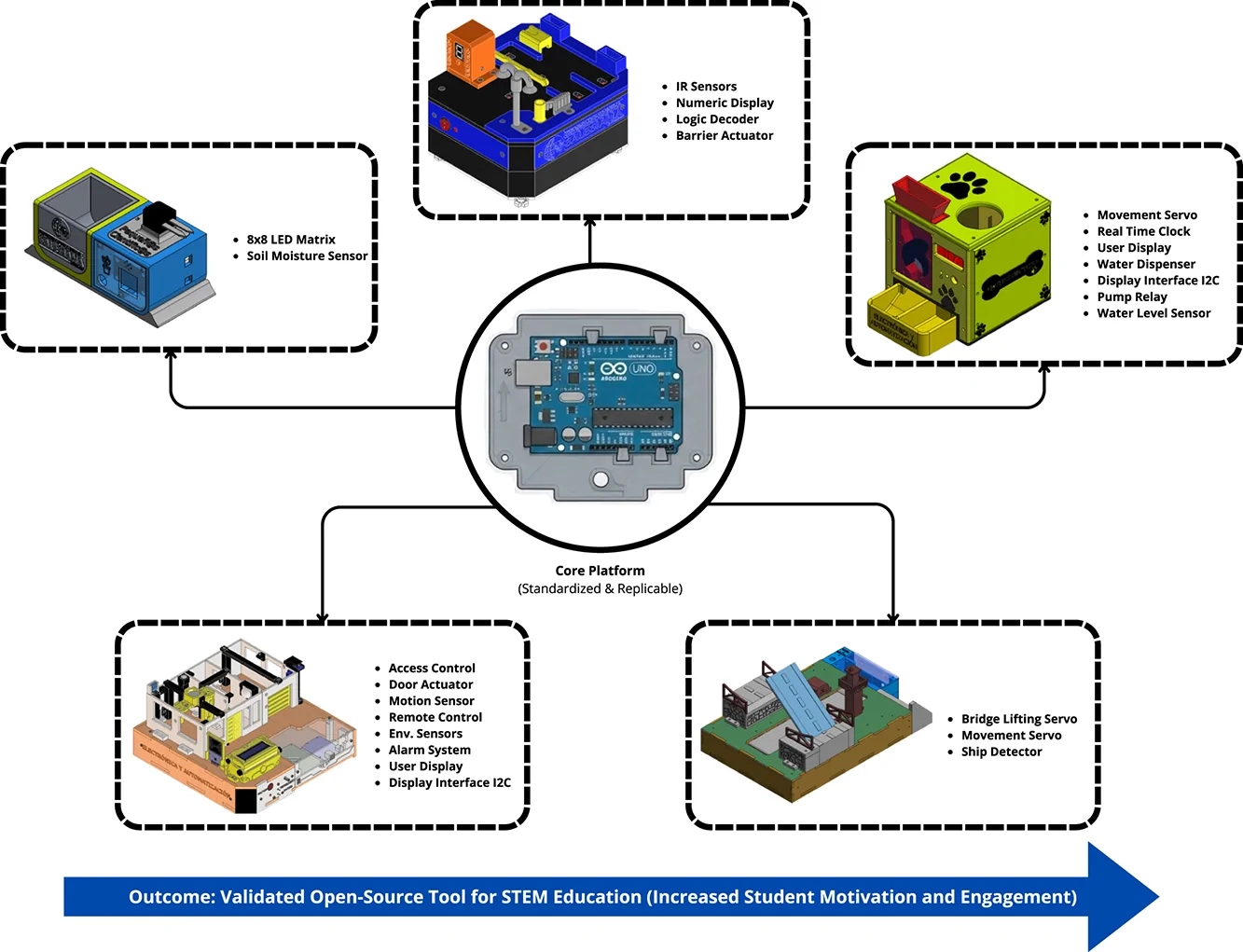

**Table:** 

Hardware name	Modular Educational Robotics Kit

Subject area	Engineering and Materials Science/Educational Hardware

Hardware type	Electrical engineering and computer science/Education

Closest commercial analog	Keyestudio Smart Home IoT Kit, Elegoo Mega Project Smart Kit, SunFounder IoT Kit, among other similar kits.

Open source license	Hardware: CC BY 4.0/Software: MIT

Cost of hardware	Approx. $548.00 USD (for the complete 5-module laboratory deployment)

Source file repository	https://data.mendeley.com/datasets/pmvdtjs2jv

## 1 Hardware in context

The integration of technology in education is increasingly prevalent. However, in general secondary education, the primary educational goal is not necessarily to train students in specific, advanced engineering competencies, but rather to foster critical thinking, problem-solving, and technological literacy—skills that are essential for the 21st century [1]. In developing regions like Ecuador, overcoming the technological gap is critical to improving global innovation indexes [2]. Furthermore, recent studies highlight the necessity of implementing active methodologies, such as the STEAM (Science, Technology, Engineering, Arts, and Mathematics) approach, to develop current competencies in students, particularly in post-pandemic or resource-constrained educational contexts [3], [4], bridging formal curriculum gaps through the deployment of interactive mobile frameworks or localized maker culture environments [5], [6]. To achieve this, the integration of computational thinking and open-source platforms like Arduino has proven highly effective for secondary education [7]. Yet, in the city of Cuenca, many middle and high schools still lack the infrastructure and resources to support these programs, making the provision of accessible robotics platforms an urgent necessity. A major barrier to adopting external international kits in these regions is the prohibitive cost of importation, which places a heavy financial burden on private institutions and becomes an almost insurmountable hurdle for public schools.

While there are numerous educational IoT and robotics kits on the market, such as the Keyestudio Smart Home IoT Kit, Elegoo Mega Project Smart Kit, and SunFounder IoT Kit, among others, they often operate as “black boxes” [8]. In this educational context, the term “black box” refers to a closed, non-modular system where the internal electronics and mechanics are hidden. Furthermore, as summarized in Table 1, mainstream commercial platforms present significant limitations regarding layout realism and module diversity. Most commercial smart-home trainers mount all peripherals arbitrarily on a flat, single-sheet Medium-Density Fibreboard (MDF) or acrylic panel, isolating components from their real-world architectural context. In contrast, the proposed kit prioritizes structural and functional realism; for instance, the home automation module implements explicit multi-room spatial tracking, interior dividing walls, and dedicated 3D-printed conduits that emulate actual residential installations. Additionally, specialized automated mechanical applications — such as the continuous-rotation conveyor drawbridge and the multi-slot absolute matrix scanning parking lot — are virtually non-existent as packaged educational kits in the commercial market, representing a major gap that this open-source design successfully fulfills.

In the academic literature, several open-source initiatives have been proposed to counter this [9]. Active project-based methodologies and low-cost physical prototyping have been heavily documented as successful strategies to spark student interest in complex scientific domains and environmental tracking [10], [11]. Furthermore, recent milestones in educational robotics showcase that physical platforms act as highly adaptable scaffolds within early training loops [12], especially when implementing realistic mathematics integration [13] and structured strategies tailored to introduce secondary students to emerging technologies and computing fundamentals [14], [15]. However, a significant gap remains between existing research and practical classroom implementation. Many previous open-source educational designs focus either on basic bare-board electronics — lacking the engaging mechanical context that keeps younger students motivated — or require advanced, expensive digital manufacturing equipment to reproduce.

**Table 1:** Comparative analysis of the proposed modular kit against mainstream commercial educational platforms.

Educational criterion	Keyestudio smart home	Elegoo project kits	SunFounder IoT	Proposed kit
Open-Source Core Ecosystem	✓	✓	✓	✓
Structural Layout Realism	Flat MDF Panel	Loose Components	Flat Acrylic Board	Architectural Realism
Modularity/Workspace Curve	Monolithic Layout	Single-Purpose	Monolithic Layout	5 Independent Stations
Automated Parking Lot Unit	×	×	×	✓
Electromechanical Drawbridge	×	×	×	✓
Localized Manufacturing Cost	High Import Cost	High Import Cost	High Import Cost	Optimized Low-Cost

The primary contribution of this work is the documentation of a proven hardware design that serves as an accessible, scalable tool for the educational community. The proposed product explicitly differs from previous research by offering a holistic, progressive difficulty curve utilizing strictly standard, off-the-shelf components (the Arduino ecosystem) combined with low-cost Fused Deposition Modeling (FDM) 3D printing. Rather than a monolithic robot, this kit is designed with a philosophy of total replicability and modularity. It guides students from building a simple environmental sensor to constructing a complex IoT-integrated home automation system using five distinct functional modules, all utilizing a single, standardized core base. This ensures that educators can implement a comprehensive robotics curriculum without relying on expensive proprietary upgrades.

## 2 Hardware description

### 2.1 General architecture

The experimentation kit is engineered based on a decoupled modular architecture centered around the Arduino UNO R3 microcontroller. The physical structural components were designed using Autodesk Fusion 360, while the electronic printed circuit boards (PCBs) and schematics were developed in Altium Designer, utilizing institutional licenses provided by Universidad Politécnica Salesiana. This professional-grade toolchain ensures high precision and reproducibility. For physical manufacturing, parts were fabricated using Fused Deposition Modeling (FDM) with Polylactic Acid (PLA) filament.

A critical feature of the hardware is its robust power infrastructure. While Module 1 (M1) is powered directly via the Arduino USB/DC jack due to its low consumption, Modules M2 through M5 share a centralized high-current power system. A 12 V-5 A DC power supply acts as the primary source. To ensure safety and operational stability, the current is distributed through four independent Step-Down (Buck) converters, each regulated to 5 V DC and protected by a dedicated fuse. This modular power approach prevents voltage drops during high-torque servo movements and protects the microcontrollers from overcurrent events, addressing key safety requirements for educational environments.

### 2.2 Description of modules

The hardware is divided into five functional modules, each focusing on specific electronic concepts ranging from basic sensing to autonomous control systems.

#### 2.2.1 Module 1: Smart Pot (environmental monitoring)

This module serves as an introductory unit for environmental sensing, featuring a dual-compartment 3D-printed chassis designed for both functional botany and electronic protection.

**Mechanical Layout:** When viewed from the front, the chassis is divided into two main sections. The left section functions as the planter, housing the soil and the biological specimen. The right section acts as the control interface; its front panel features a dedicated mounting slot for the 8 × 8 LED Matrix and a circular orifice for the main power toggle switch. The internal cavity of this right section houses the Arduino UNO, which is vertically mounted to the internal partition wall directly behind the LED Matrix to optimize space and cable routing. To facilitate sensor integration, a cable-routing orifice is situated at the top of the chassis, allowing the soil moisture sensor leads to reach the planter compartment while keeping the electronics isolated from moisture.

**Electronic Integration and Assembly:** For structural assembly, standardized M3 screws and nuts were used, with lengths ranging between 8 mm and 12 mm. To facilitate electrical interconnections, a simple custom PCB was developed featuring pin headers to manage power distribution between the power switch, the moisture sensor, the Arduino, and the 8 × 8 LED matrix. While power is centralized through this PCB to simplify wiring, data communication remains direct: the FC-28 sensor’s analog signal and the 8 × 8 matrix’s SPI lines are connected directly to the Arduino’s I/O pins to ensure signal integrity. This module relies on the Arduino’s internal power regulation, as it lacks high-torque actuators.

**Visual Feedback Mechanism:** The system provides intuitive feedback through the 8 × 8 LED matrix using binary-mapped icons. When the sensor detects sufficient moisture, a “happy face” icon is displayed to indicate optimal conditions. Conversely, when moisture levels drop below the defined threshold, the display switches to a “sad face” icon, signaling the need for irrigation. This visual signaling, as illustrated in Fig. 1, simplifies complex sensor data into an immediate, empathetic response for the student.

Fig. 1Smart Flower Pot: Detailed layout showing the electronic compartment (right) and the planting area (left). The LED matrix provides real-time visual feedback using hydration-status icons.Fig. 1

**(a):**
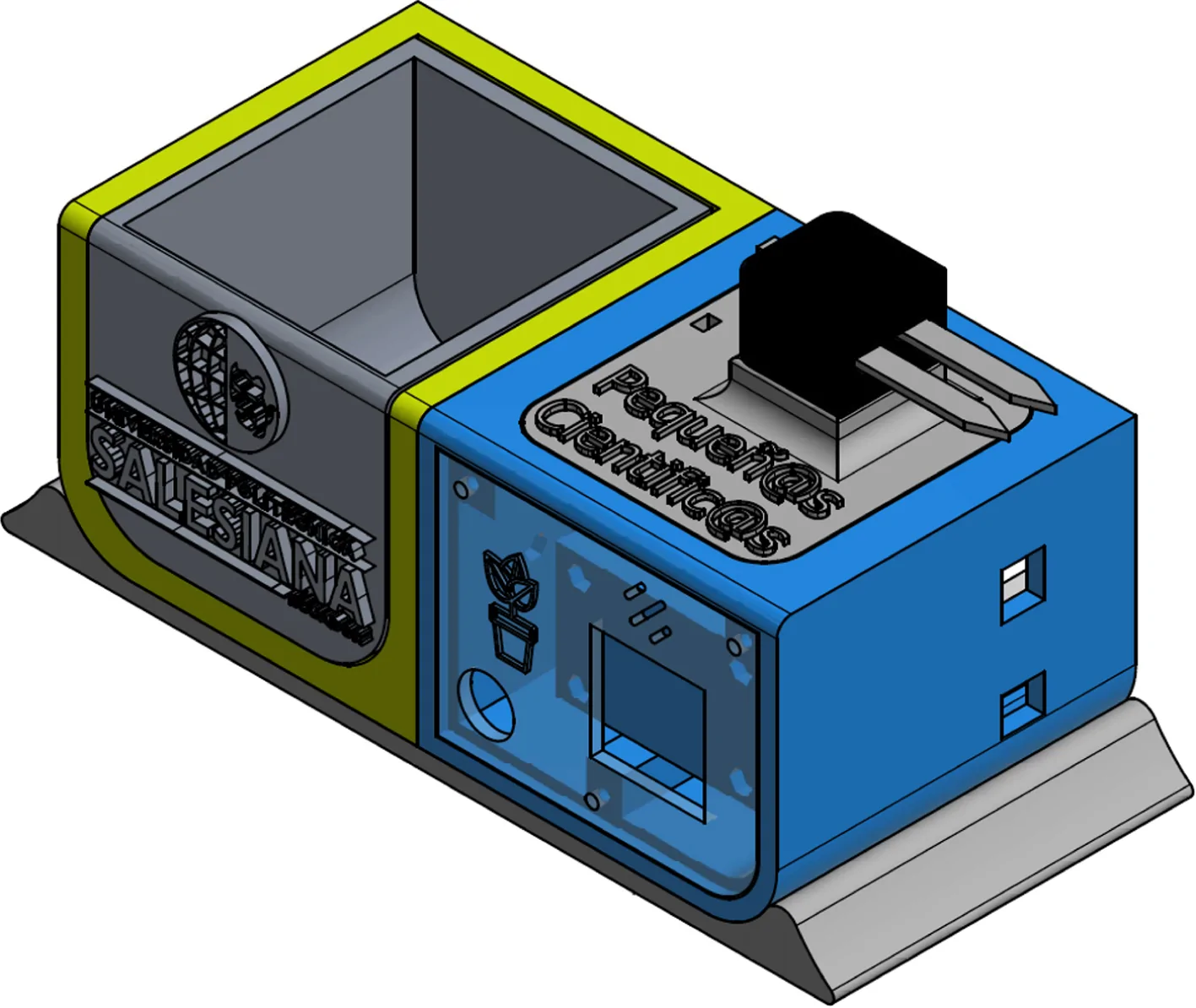
Smart Pot (prototype).

**(b):**
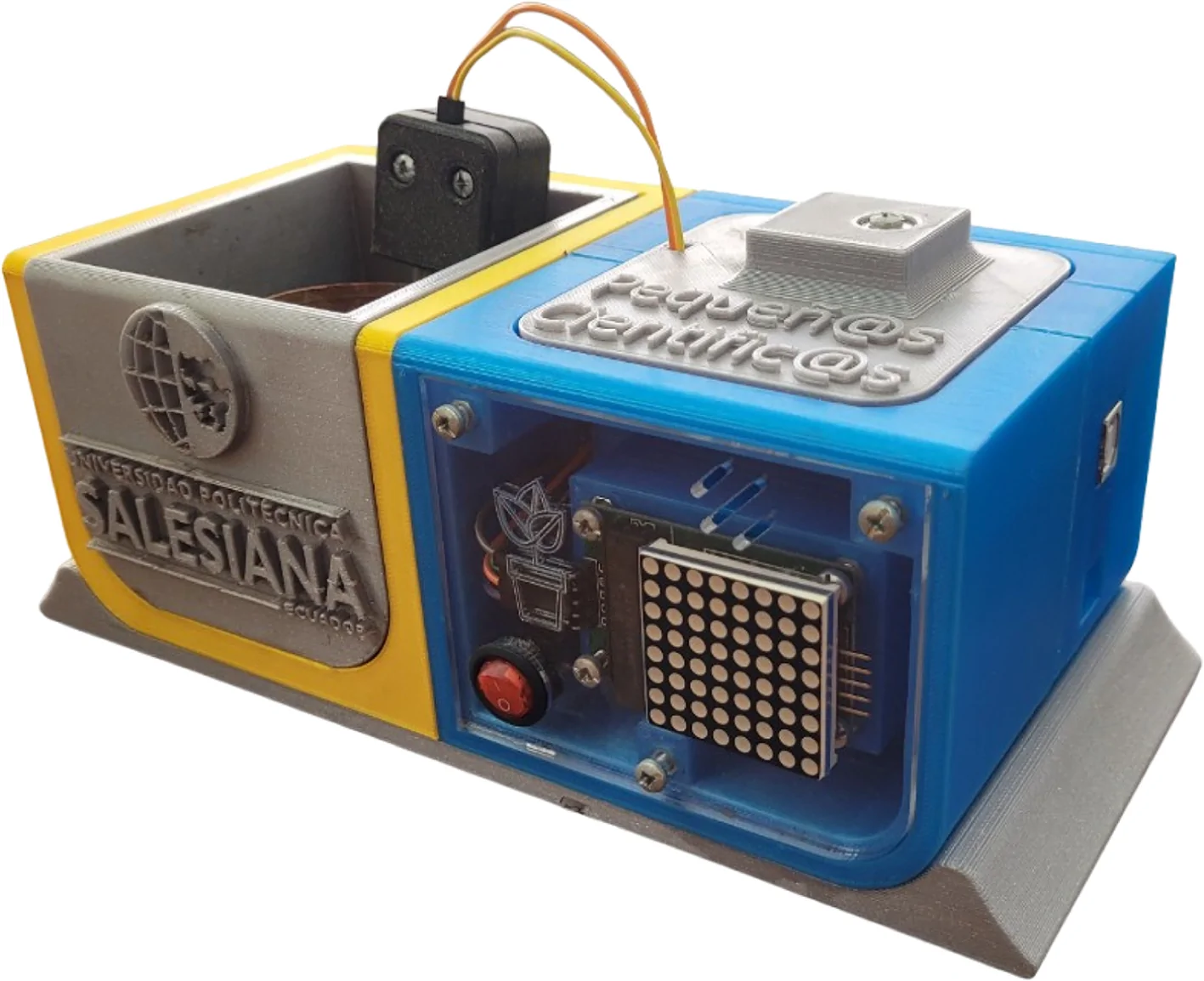
Smart Pot (real implementation).

#### 2.2.2 Module 2: Automated Parking System

This module simulates a real-world smart parking infrastructure. It focuses on integrating optical sensing, mechanical actuation, and digital logic circuits within a comprehensive 3D-printed environment.

**Mechanical Layout and Assembly:** The PLA 3D-printed chassis is designed as a dual-level structure. Structural integrity between the layers and components is achieved using standardized M3 screws with lengths ranging from 15 mm to 25 mm. The top surface emulates the parking lot environment, featuring clearly defined parking slots, a roadway, and a printed light pole equipped with two decorative LED diodes. The entrance is guarded by a physical barrier actuated by an SG90 micro-servo motor. To mimic real parking systems, TCRT5000 infrared sensors are strategically positioned: one at the entrance gate to detect arriving vehicles, and additional sensors embedded within each parking slot to monitor individual occupancy. The 7-segment display is prominently mounted to be easily visible to the user.

**Electronic Integration and Power Management:** To maintain a clean and realistic upper surface, all complex wiring is concealed in a covered compartment beneath the parking platform. This lower section houses the Arduino UNO and a custom-designed Printed Circuit Board (PCB), which routes the sensor arrays and houses the 74LS48 BCD-to-7-segment decoder. Power is supplied from the external 12 V-5 A centralized source. Crucially, this module utilizes its own dedicated step-down buck converter, regulating the input to a stable 5 V. While the current draw of the SG90 and LEDs is relatively moderate, this isolated regulation ensures absolute operational stability and protects the logic circuits from any potential voltage spikes.

**Operation and Dynamic Feedback:** The system operates as a finite state machine. When a vehicle approaches, the entrance IR sensor detects it, and the Arduino commands the SG90 servo to lift the barrier. As vehicles occupy or vacate the internal parking slots, the embedded IR sensors send real-time data to the microcontroller. The Arduino calculates the available capacity and updates the 7-segment display via the 74LS48 decoder, providing immediate, dynamic numerical feedback identical to a commercial automated parking facility, as illustrated in Fig. 2.

Fig. 2Automated parking system: The physical implementation (right) hides the custom PCB and Arduino within the lower chassis, exposing only the sensors, actuator, and the 7-segment display.Fig. 2

**(a):**
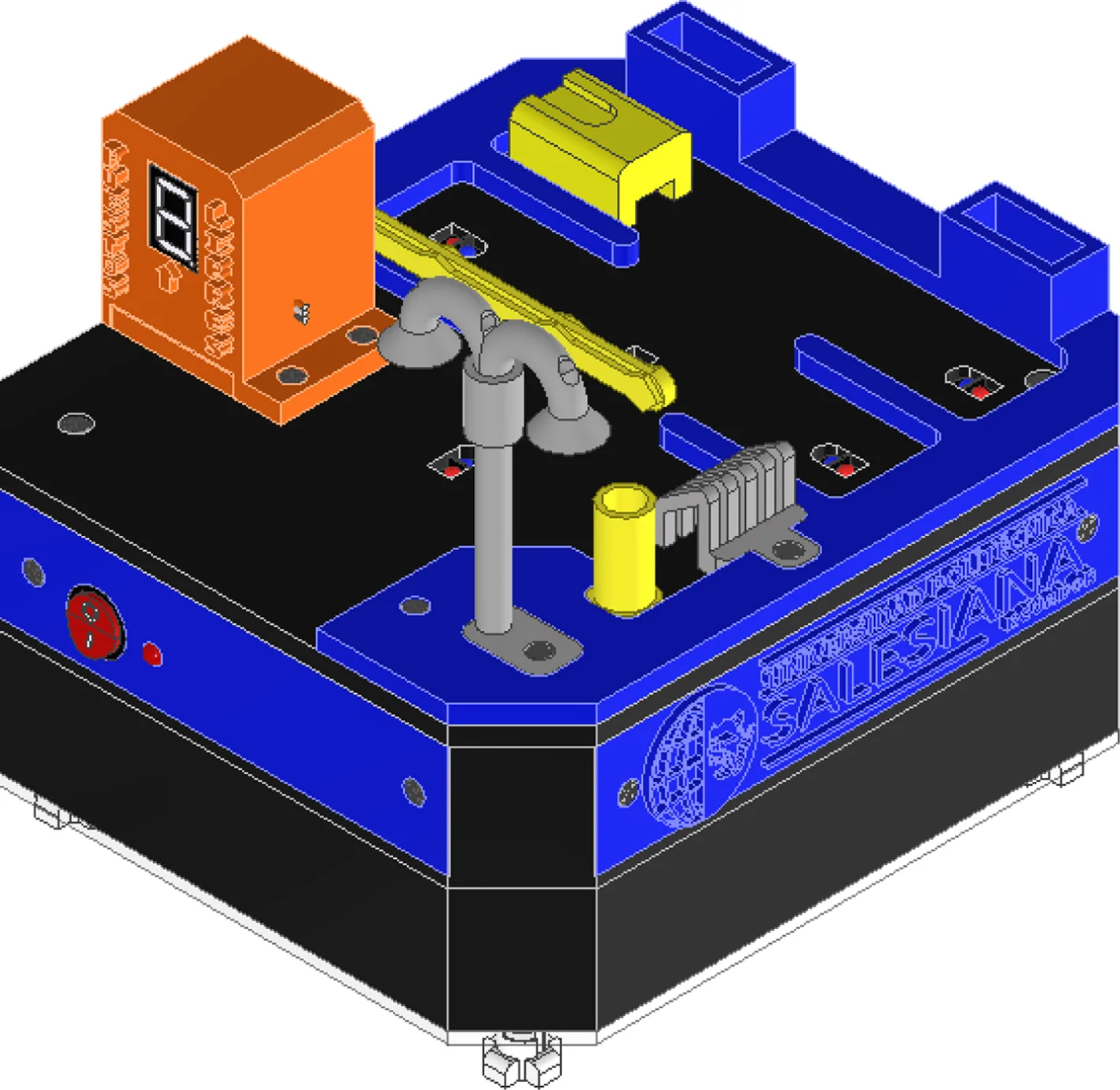
Automated parking (prototype).

**(b):**
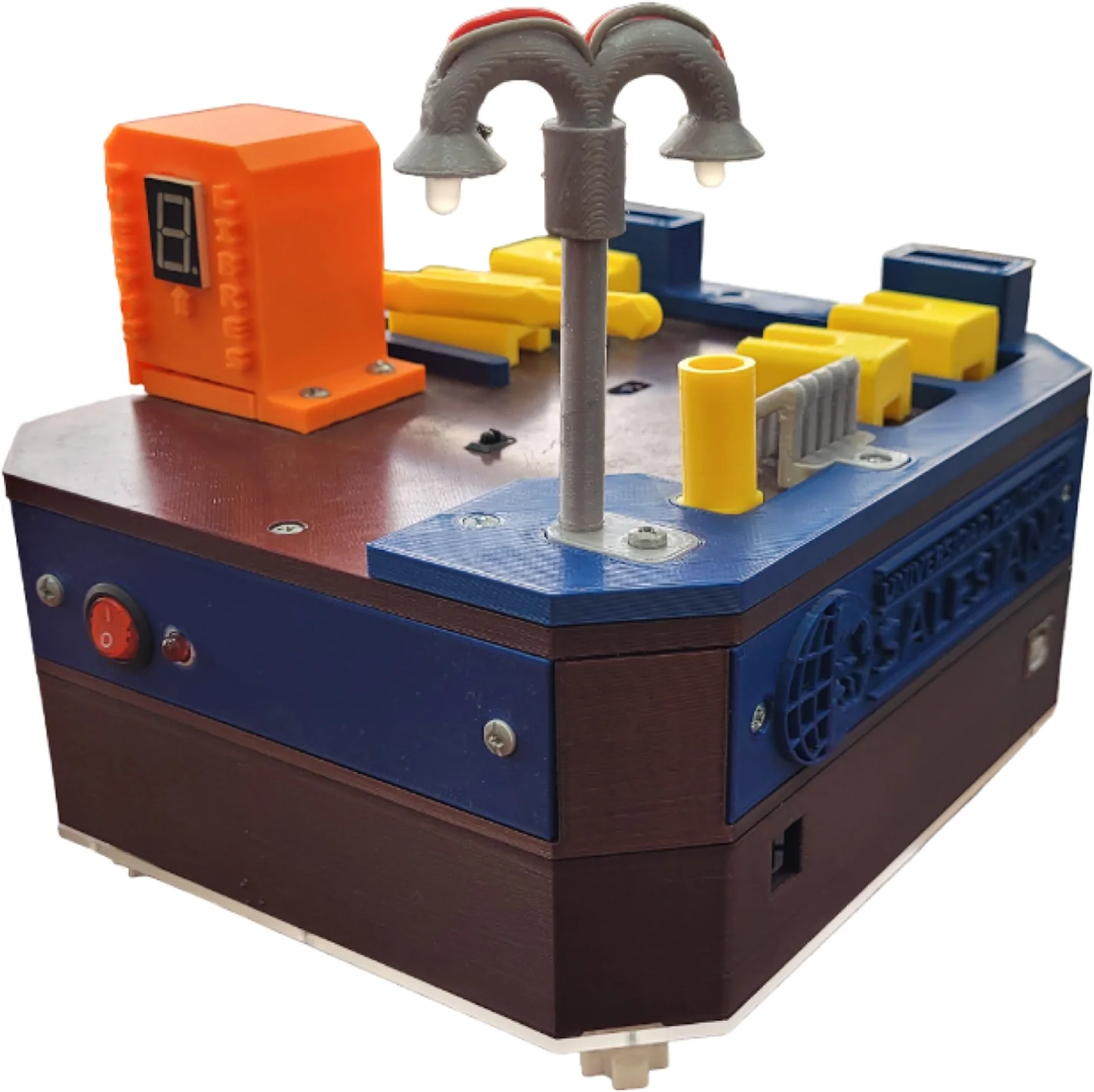
Automated parking (real implementation).

#### 2.2.3 Module 3: Drawbridge Mechanism (electromechanical integration)

This module is the most mechanically complex unit, designed to demonstrate high-torque actuation, optical barriers, and hybrid material manufacturing (MDF and 3D printing).

**Mechanical Layout and Hybrid Structure:** Given the large scale of this module (481 × 312 mm), a hybrid manufacturing approach was adopted. The main base is fabricated from MDF (Medium-Density Fibreboard), providing the structural rigidity necessary to support the dynamic loads of the bridge. This base is covered with artificial grass for aesthetic realism. The module features two distinct systems:

• **River Simulation (Conveyance System):** To emulate water flow beneath the bridge, a sliding blue canvas belt is used. This belt is driven by a two-roller system. The active roller is powered by a 360° continuous rotation servo (MG996R) coupled with a high-precision XIKE 6004-2RS double-rubber bearing. The passive roller utilizes two identical bearings to ensure smooth motion and prevent belt misalignment.
• **Drawbridge Mechanism:** The bridge deck features a roadway design that elevates to allow “vessels” to pass. It is actuated by a high-torque DS3218 (20 kg-cm) servo motor mounted horizontally. The mechanical design defines a starting angle of 48° and a final elevation of 90° (a 42° stroke), ensuring realistic movement and preventing structural strain.

**Power and Operational Stability:** Following the kit’s centralized power strategy, this module is fed by the primary 12 V-5 A source. To address the high current demands of the electromechanical actuators, a dedicated high-efficiency Step-Down (Buck) converter is used to regulate the voltage to 8 V DC (supporting peaks up to 3 A). While the DS3218 servo is rated for 20 kg-cm, it was selected not for its maximum lifting capacity — as the 3D-printed bridge deck is relatively light — but to guarantee optimal performance, durability, and smooth motion under continuous use. This “oversizing” strategy, combined with the Buck converter’s ability to handle the 2.5 A stall current peaks of the motors, ensures that the microcontroller remains isolated from electrical noise and voltage sags, providing a stable learning environment even during simultaneous bridge and river-belt operation, as shown in Fig. 3.

Fig. 3Drawbridge Mechanism.Fig. 3

**(a):**
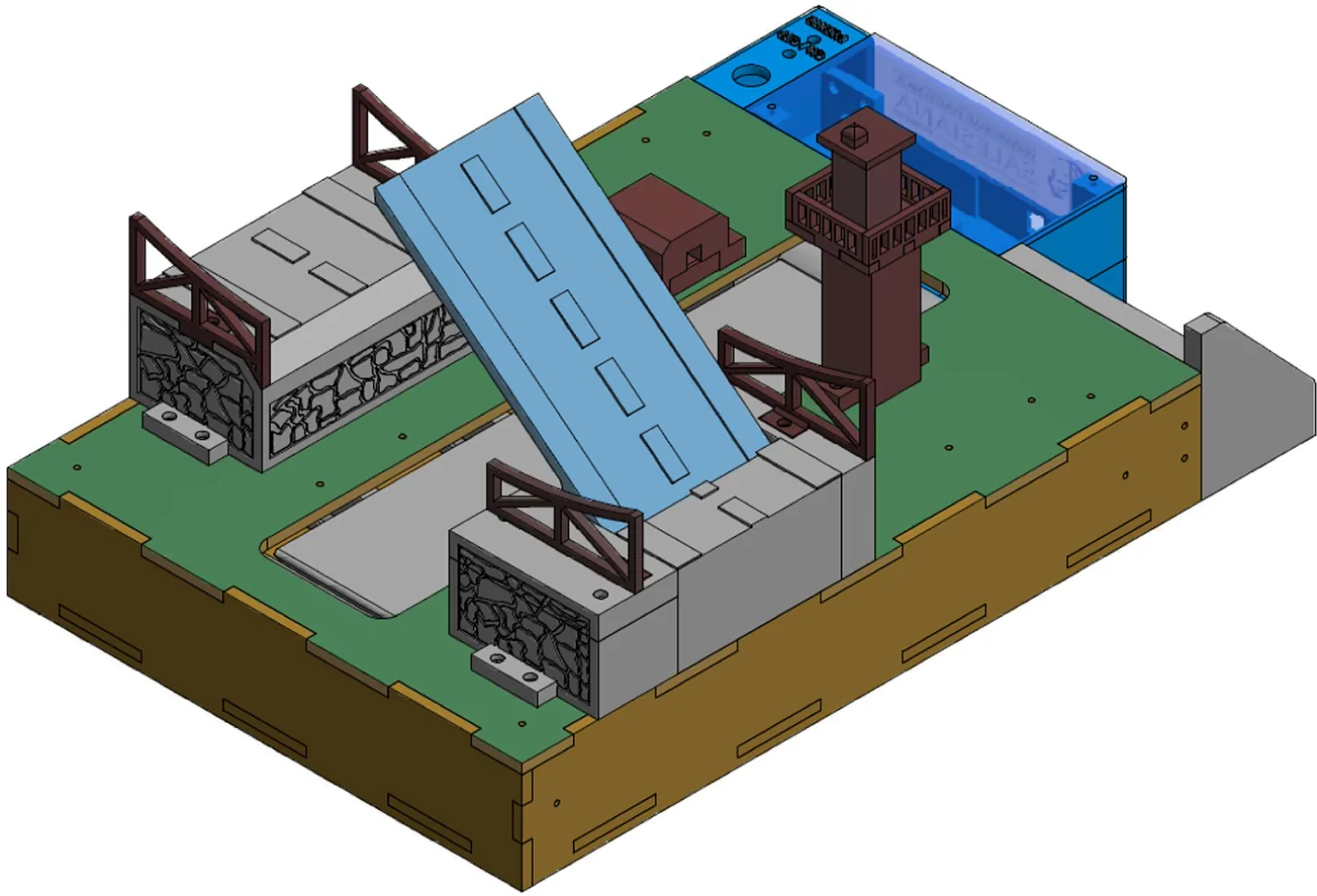
Drawbridge (prototype).

**(b):**
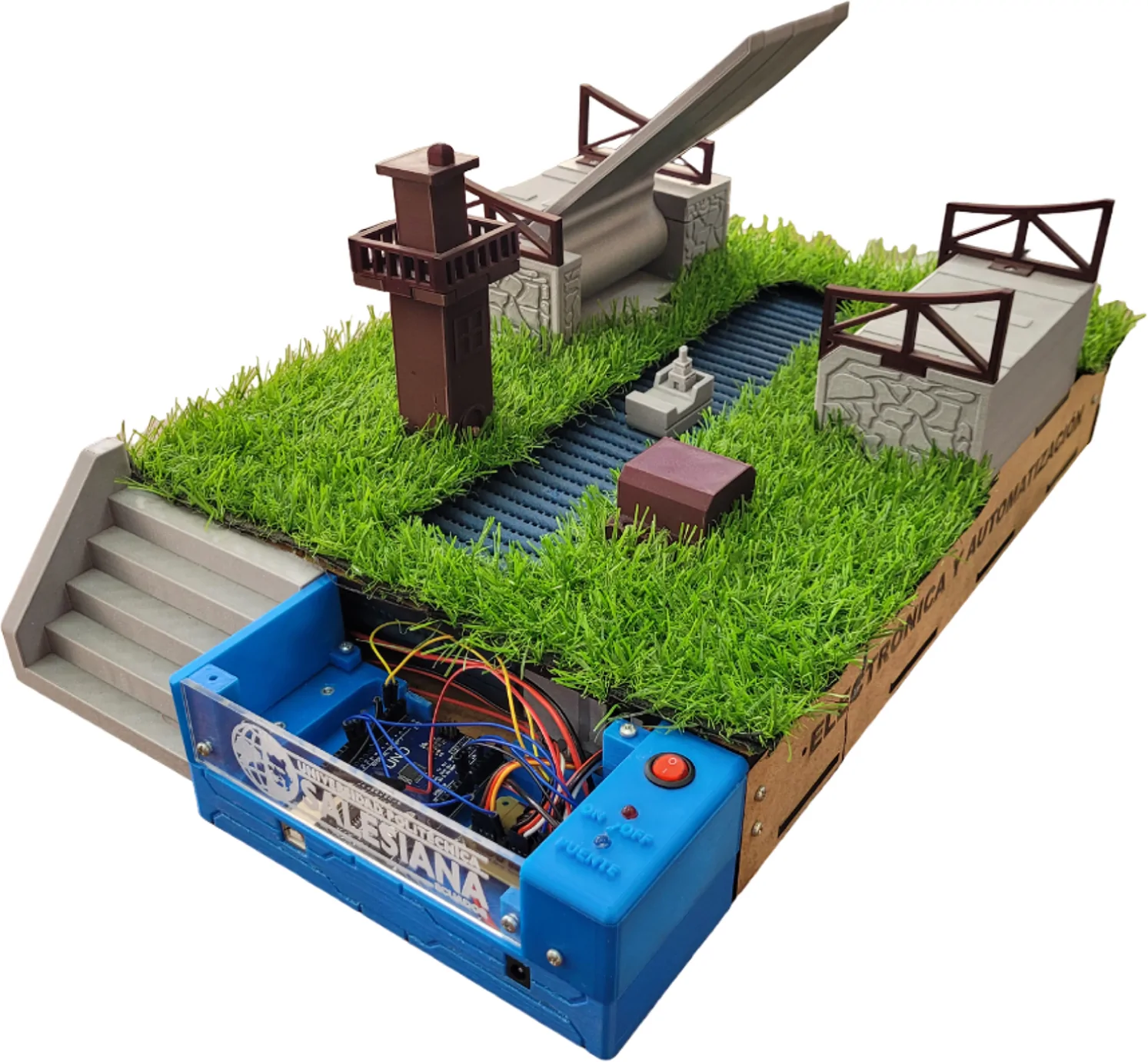
Drawbridge (real implementation).

#### 2.2.4 Module 4: Automated food dispenser (timed automation and fluid control)

This module represents a complex system (212 × 208 × 213 mm) that integrates scheduled dry-food dispensing and automated water replenishment using real-time monitoring.

**Mechanical Layout and Material Hybridization:** The dispenser features a 3D-printed PLA chassis with a strategic hybrid design. A transparent acrylic front panel was implemented to allow students to visually monitor the dispensing mechanism and internal storage levels. The module is divided into three functional zones:

• **Solid Food Mechanism:** A custom-designed rotary wheel acts as the gate valve. It is coupled to a high-torque DS3218 (20 kg-cm) to a 180° servo motor. The dispensing sequence involves a controlled stroke from an initial position of 30° (closed) to 170° (open), precisely timed to release a specific volume of food before returning to the standby position.Although over-dimensioned for the required load, this servo was selected to ensure high reliability, preventing jams and guaranteeing smooth operation during long-term use.
• **Fluid Management System:** A dedicated base holds a standard water reservoir. A 5 V pump, connected via a 5 mm transparent hose, handles the water transfer. The system utilizes a water level sensor to trigger the pump via a Relay Module, ensuring the dish remains filled.On the right side of the module, a dedicated housing holds a hermetic plastic reservoir (adapted from a commercial airtight container). The lid was specifically modified to provide a secure, leak-proof entry point for the 5 mm transparent suction hose.
• **Electronics Compartment:** To protect the logic circuits from potential spills, the Arduino UNO and the custom-designed PCB are housed in an isolated rear compartment, providing high stability and ease of access for maintenance.

**Electronic Integration and Control Logic:** The control architecture is centered on a DS3231 Real-Time Clock (RTC), which provides high-precision timekeeping even during power-off states due to its integrated battery. A 16 × 2 LCD screen with an I2C interface displays the real-time date and hour, alongside a front-facing user interface consisting of an ON/OFF button and LED status indicators (Power and Water Level). For structural integrity, M3 screws of 10, 15, and 20 mm were used throughout the assembly.

**Operation and Power Management:** In this module, the centralized power source is regulated to a stable 5 V DC via a dedicated Step-Down converter. This provides consistent voltage for the relay-driven water pump, the servo motor, and the logic circuits. The automation follows a scheduled routine: the Arduino compares the RTC data against programmed thresholds (e.g., breakfast, lunch, and dinner). When a match is detected, the dispensing sequence is triggered. Concurrently, the water level sensor continuously monitors the hydration status, activating the relay as a closed-loop response to depletion. This provides consistent voltage for the relay-driven water pump, the 20 kg-cm servo motor, and the logic circuits, as depicted in Fig. 4.

Fig. 4Automated food dispenser.Fig. 4

**(a):**
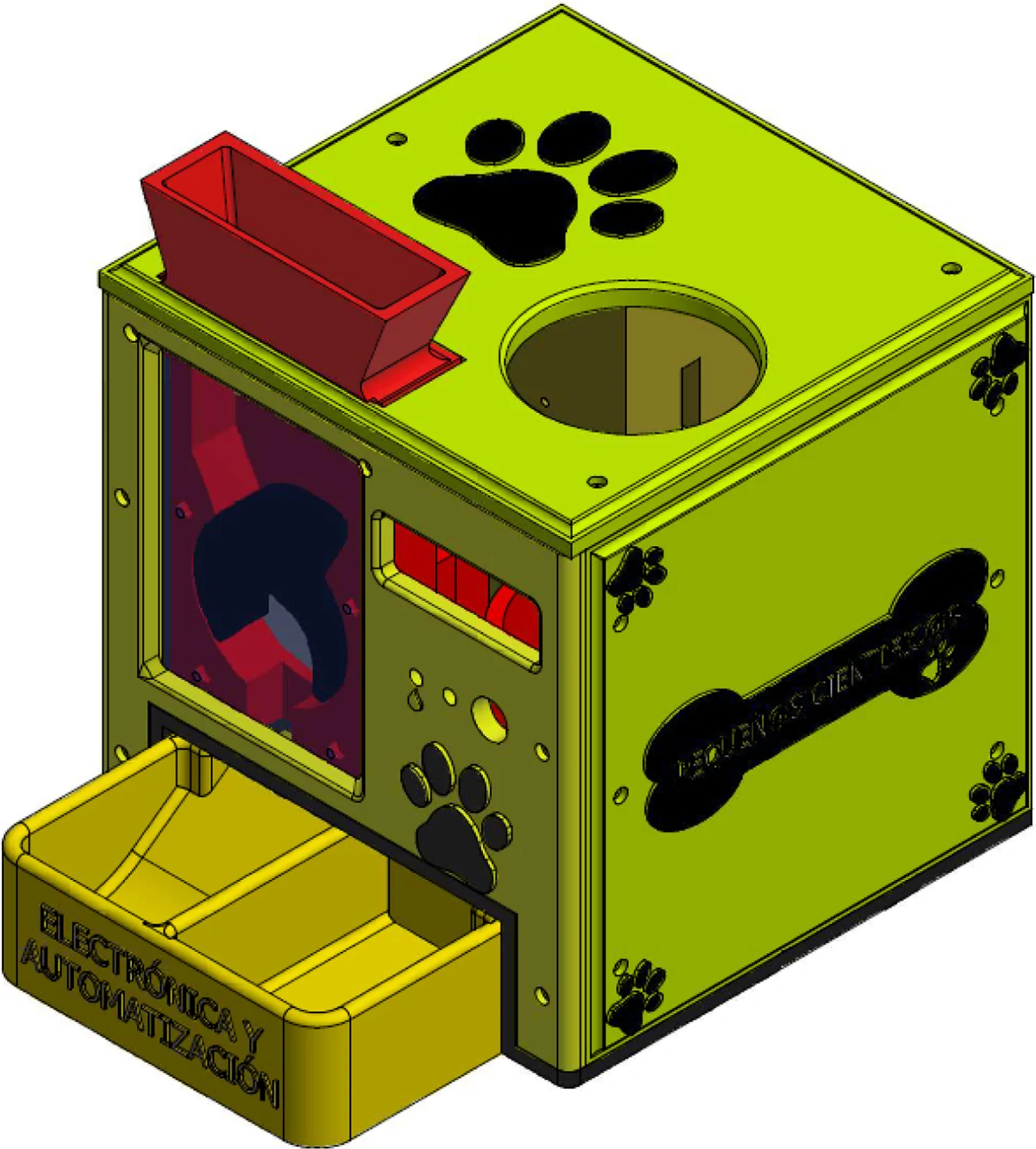
Food dispenser (prototype).

**(b):**
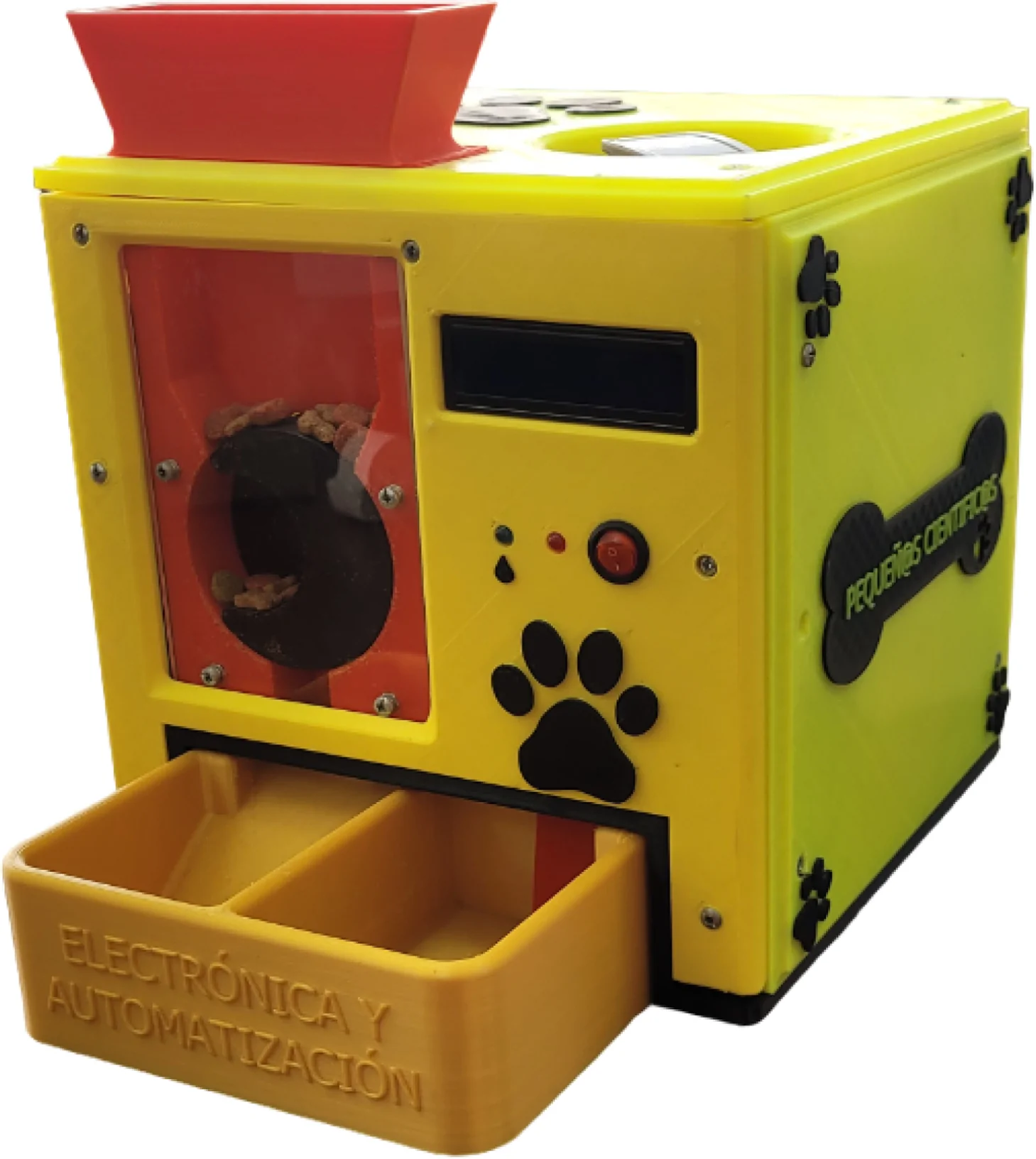
Food dispenser (real implementation).

#### 2.2.5 Module 5: Home automation system (comprehensive IoT and domotics)

The final module is the capstone of the kit, representing a high-complexity domotic system (285×278.25 mm) designed to introduce students to security, environmental control, and human-machine interfaces (HMI).

**Mechanical Layout and Educational Transparency:** The module is built upon a hybrid MDF and PLA structure. A sturdy MDF base provides the main structural support and cable management channels, while the house walls are 3D-printed to emulate a single-story residence.

• **Visual Access:** To maximize educational value, the design omits a roof, allowing students to observe the internal spatial distribution and sensor placement. Furthermore, the Arduino UNO is located at the front of the base, protected by a transparent acrylic cover, showcasing it as the “brain” of the system.
• **Spatial Distribution:** The house features a functional garage on the right (actuated by an SG90 servo) and a main entrance on the left (actuated by a second SG90 servo). The RFID reader is positioned at the front “patio” in a dedicated 3D-printed housing for easy access. To avoid signal interference, the IR receiver is mounted at the top of the garage, while the PIR motion sensor is installed above the main entrance, mimicking real-world installation heights.

**Electronic Integration and Control Interface:** This module integrates 11 distinct electronic components. Wiring is meticulously managed through internal black 3D-printed conduits that hide the cables while maintaining aesthetic realism. The control interface, located on the front panel, includes an I2C LCD screen, a main power switch, and two “toggle” switches to manually enable the alarm system (buzzer) and the ventilation system. A dedicated potentiometer (dimmer) allows for analog control of the internal SMD LED lighting blocks.

**Operation and Power Management:** Given the high density of peripherals — including a 5 V DC fan motor — this module utilizes a dedicated Step-Down converter regulated to a stable 5 V DC. The system operates through multiple logic layers:

• **Access Control:** Biometric-style entry is simulated via the RC522 RFID module; authorized tags trigger the servo-actuated doors. Additionally, an IR remote control provides a secondary access method for the garage and the principal door.
• **Environmental Control:** A DHT11 sensor monitors temperature and humidity. When a predefined threshold is reached (or via manual override), the 5 V DC motor activates to simulate climate control.
• **Security:** A PIR sensor detects proximity at the entrance, triggering a rear-mounted buzzer (active/passive) to emulate a real-world alarm system. Unless the alarm is turned off using the switch provided for that purpose.

This complete physical layout and hardware distribution are illustrated in Fig. 5.

Fig. 5Automated house system.Fig. 5

**(a):**
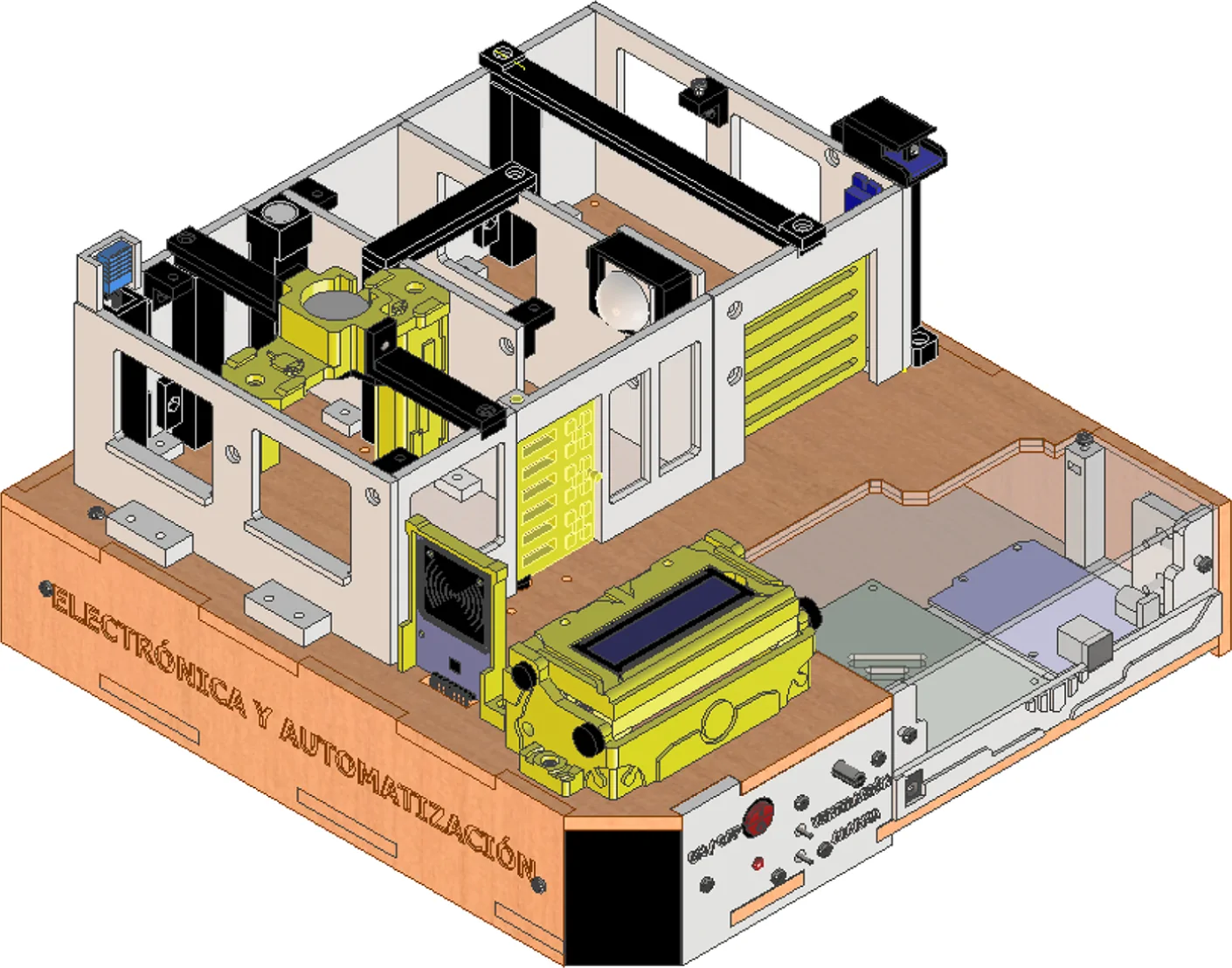
Automated house (prototype).

**(b):**
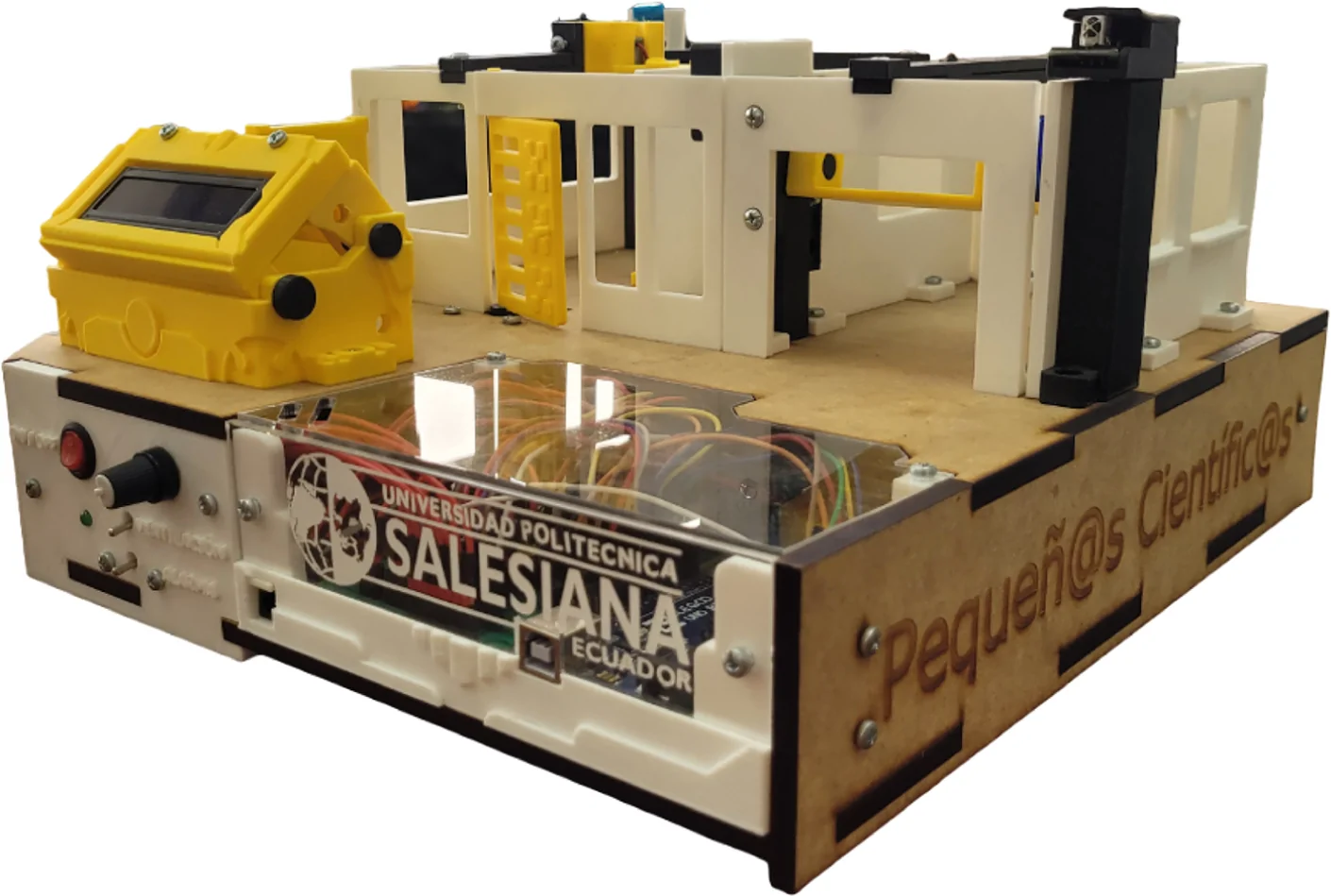
Automated house (real implementation).

## 3 Design files summary

The complete open-source design files are available for download at the Mendeley Data repository: https://data.mendeley.com/datasets/pmvdtjs2jv. These files ensure full replicability across all mechanical, electronic, firmware, and documentation layers, as summarized in Table 2.

**Table 2:** Summary of design files organized by functional module.

File name	File type	Open source license	Description	Location of the file
**Module 1: Smart Pot**				
Smart Pot 3D Design.step	CAD Model	CC BY 4.0	Assembly file containing the dual-compartment chassis (planter and electronics base) with mounting slots for components.	Repository/Module 1 Smart Pot/Mechanical
Smart Pot Animation.wmv	Animation	CC BY 4.0	Visual assembly sequence of the 3D-printed chassis parts.	Repository/Module 1 Smart Pot/Mechanical
Smart_Pot_Code.ino	Firmware	MIT	Arduino firmware handling FC-28 analog sampling and icon mapping for the 8 × 8 LED matrix feedback.	Repository/Module 1 Smart Pot/Firmware /Smart_Pot_Code
Smart Pot PCB.PrjPcb	Altium Project	CC BY 4.0	Electronic design files for the single-layer custom PCB and schematic routing.	Repository/Module 1 Smart Pot/Electronics/Smart Pot PCB Altium Source
Materials.pdf	Document	CC BY 4.0	Bill of Materials specifically for the electronic components of the Smart Pot module.	Repository/Module 1 Smart Pot/Electronics
1. Smart Pot.pdf	User Guide	CC BY 4.0	Comprehensive manual including wiring diagrams and operational procedures for Kit 1.	Repository/Module 1 Smart Pot/Documentation

**Module 2: Automated Parking**				
Automated Parking 3D Design.step	CAD Model	CC BY 4.0	Assembly file of the complete 3D design structure.	Repository/Module 2 Automated Parking /Mechanical
Automated Parking Animation.wmv	Animation	CC BY 4.0	Instructional video demonstrating the mechanical assembly of the automated parking structure.	Repository/Module 2 Automated Parking/Mechanical
Automated_Parking _Code.ino	Firmware	MIT	C++ Firmware for parking counter state-machine logic.	Repository/Module 2 Automated Parking/Firmware /Automated_Parking_Code
Automated Parking PCB.PrjPcb	Altium Project	CC BY 4.0	PCB design project files (Altium Designer).	Repository/Module 2 Automated Parking/Electronics/Automated Parking PCB Altium Source
Materials.pdf	Document	CC BY 4.0	Bill of Materials (BOM) for Kit 2 PCB.	Repository/Module 2 Automated Parking/Electronics
2. Automated Parking.pdf	User Guide	CC BY 4.0	Guide for the use of Kit 2, presenting its components and general implementation.	Repository/Module 2 Automated Parking /Documentation

**Module 3: Drawbridge**				
Drawbridge 3D Design.step	CAD Model	CC BY 4.0	Assembly file of the complete 3D design and mechanical linkages.	Repository/Module 3 Drawbridge/Mechanical
Drawbridge Animation.wmv	Animation	CC BY 4.0	Assembly animation detailing the dual-actuator mechanism for Kit 3.	Repository/Module 3 Drawbridge/Mechanical
Drawbridge_Code.ino	Firmware	MIT	C++ Firmware for continuous river motor and high-torque lifting servo control logic.	Repository/Module 3 Drawbridge/Firmware /Drawbridge_Code
Drawbridge PCB.PrjPcb	Altium Project	CC BY 4.0	PCB design project files for high-torque motor power regulation.	Repository/Module 3 Drawbridge/Electronics/Drawbridge PCB Altium Source
Materials.pdf	Document	CC BY 4.0	Bill of Materials (BOM) for Kit 3 PCB.	Repository/Module 3 Drawbridge/Electronics
3. Drawbridge.pdf	User Guide	CC BY 4.0	Guide for the use of Kit 3, presenting its components and general implementation.	Repository/Module 3 Drawbridge/Documentation

**Module 4: Automated Food Dispenser**				
Automated Food Dispenser 3D Design.step	CAD Model	CC BY 4.0	Assembly file of the complete 3D design including volumetric hopper.	Repository/Module 4 Automated Food Dispenser/Mechanical
Automated Food Dispenser Animated.wmv	Animation	CC BY 4.0	Exploded view of the 3D-printed elements and fluid container layout.	Repository/Module 4 Automated Food Dispenser/Mechanical

Automated_Food_Dispenser _Code.ino	Firmware	MIT	C++ Firmware for RTC scheduling, LCD interfacing, and closed-loop pump logic.	Repository/Module 4 Automated Food Dispenser/Firmware /Automated_Food _Dispenser_Code
Automated Food Dispenser PCB.PrjPcb	Altium Project	CC BY 4.0	PCB design project files (Altium Designer).	Repository/Module 4 Automated Food Dispenser/Electronics/Automated Food Dispenser PCB Altium Source
Materials.pdf	Document	CC BY 4.0	List of structural and electronic components for this specific module.	Repository/Module 4 Automated Food Dispenser/Electronics
4. Automated Food Dispenser.pdf	User Guide	CC BY 4.0	Guide for the use of Kit 4, presenting its components and general implementation.	Repository/Module 4 Automated Food Dispenser/Documentation

**Module 5: Home Automation**				
Home Automation (Domotics) 3D Design.step	CAD Model	CC BY 4.0	STEP file for the architectural house structure and cable conduits.	Repository/Module 5 Home Automation/Mechanical
Home_Automation _Code.ino	Firmware	MIT	C++ Firmware for multi-peripheral domotics integration (RFID, PIR, DHT11).	Repository/Module 5 Home Automation/Firmware /Home_Automation _Code
Home Automation (Domotics) PCB.PrjPcb	Altium Project	CC BY 4.0	High-density PCB design project files (Altium Designer).	Repository/Module 5 Home Automation/Electronics /Home Automation (Domotics) PCB Altium Source
Materials.pdf	Document	CC BY 4.0	Bill of Materials (BOM) for Kit 5 PCB.	Repository/Module 5 Home Automation/Electronics
5. Home Automation (Domotics).pdf	User Guide	CC BY 4.0	Guide for the use of Kit 5, presenting its components and general implementation.	Repository/Module 5 Home Automation/Documentation

**Shared Core Dependencies**				
Adafruit_Sensor -master.zip	Library	Apache 2.0	Unified sensor library baseline for Arduino (v1.1.5).	Repository/Shared Libraries
DHT_sensor _library.zip	Library	MIT	Precision timing driver for DHT11 and DHT22 telemetry data (v1.4.7).	Repository/Shared Libraries
IRremote.zip	Library	MIT	Multi-protocol handheld IR telemetry tokens capture driver (v4.4.0).	Repository/Shared Libraries
LedControl.h (v1.0.6).zip	Library	MIT	Low-level register manipulation driver for MAX7219 8 × 8 matrix (v1.0.6).	Repository/Shared Libraries
LiquidCrystal _I2C.zip	Library	MIT	Specialized driver managing alphanumeric screens over I2C (v1.1.2).	Repository/Shared Libraries
MFRC522.zip	Library	MIT	SPI core abstraction library for MFRC522 RFID communication (v1.4.12).	Repository/Shared Libraries

## 4 Bill of materials

The total estimated cost for the complete five-module kit is approximately $548.00 USD. This budget reflects the logistical and economic reality of developing high-quality educational hardware in Ecuador. The procurement strategy was twofold: specialized microcontrollers and precision sensors were imported directly through international open-market distributors (such as Adafruit and SparkFun), while structural materials and discrete electronic components were acquired through specialized global and national suppliers (such as DigiKey, Mouser, and local technical providers).

It is important to highlight that a significant portion of the budget was allocated to local technical services, specifically high-quality PCB fabrication for the custom Altium designs and precision laser cutting for the MDF and acrylic components. In the Ecuadorian economic context, these specialized manufacturing processes represent a higher investment compared to mass-produced alternatives, but they were essential to ensure the kit’s structural durability and professional finish. All design files, including Altium PCB projects and Fusion 360 models, are available at the Mendeley Data repository: https://data.mendeley.com/datasets/pmvdtjs2jv. The complete itemized cost breakdown for all structural, electronic, and active peripheral components is fully detailed in Table 3.

**Table 3:** Detailed Bill of Materials (BOM) organized by Functional Module.

Designator	Component/Model	Material type	Source of materials	Qty	Unit cost ($)	Total cost ($)
**Module 1: Smart Flower Pot (Environment)**						
Arduino	Arduino UNO R3 Core	Semiconductor	Adafruit/Retailers	1	$28.00	$28.00
JP3 (Sen_agua)	FC-28/Capacitive Probe Soil Moisture Sensor	Semiconductor	SparkFun/Online	1	$3.00	$3.00
MAX7219	MAX7219 8 × 8 LED Module Matrix Display	Semiconductor	SparkFun/Online	1	$4.00	$4.00
JP2 (Switch)	Manual Toggle Switch Power Control	Non-specific	DigiKey/Mouser	1	$1.00	$1.00
PCB1	Altium Single-layer Custom PCB	Composite	Custom PCB Bureau	1	$5.00	$5.00
STRUCT1	PLA Pot Chassis 3D Structure	Polymer	Self-printed (FDM)	1	$10.00	$10.00
7805, D1, R1–R2...	Discrete Electronics Pack (7805, D1, 470μF, 100nF, R1–R2, Headers)	Semiconductor	DigiKey/Mouser	1	$1.50	$1.50

**Module 2: Automated Parking**						
Fuente_Arduino	Arduino UNO R3 Core	Semiconductor	Adafruit/Retailers	1	$28.00	$28.00
Sen1–Sen4, Sen_ing, Sen_sal	TCRT5000 IR Barrier Module Sensors	Semiconductor	SparkFun/Online	2	$1.00	$2.00
DS3	7-Segment Blue CC Display (LED7SEG-1)	Semiconductor	DigiKey/Mouser	1	$1.50	$1.50
74-47/48	74LS48/7447 BCD Decoder IC (DIP16)	Semiconductor	DigiKey/Mouser	1	$1.00	$1.00
Barrera	Micro Servo SG90 (9g) Barrier Actuator	Non-specific	Adafruit/Online	1	$3.00	$3.00
REG1	LM2596 Step-Down Buck Module	Semiconductor	SparkFun/Online	1	$2.50	$2.50
PCB2	Altium Single-layer Custom PCB	Composite	Custom PCB Bureau	1	$8.00	$8.00
D1	1N4004 Rectifier Diode (D1N4004)	Semiconductor	DigiKey/Mouser	1	$0.20	$0.20
Jack DC1	14 × 9mm Jack DC Panel Mount	Non-specific	DigiKey/Mouser	1	$1.00	$1.00
LED1, LED2, LED3	3 mm Red Signaling LEDs	Semiconductor	DigiKey/Mouser	3	$0.10	$0.30
R1–R10	330Ω 1/4 W Through-hole Resistors	Semiconductor	DigiKey/Mouser	10	$0.05	$0.50
R11, R13, R15, R17, R19, R21	220Ω 1/4 W Sensor Pull-up Resistors	Semiconductor	DigiKey/Mouser	6	$0.05	$0.30
R12, R14, R16, R18, R20, R22	5.1kΩ 1/4 W Logic Base Resistors	Semiconductor	DigiKey/Mouser	6	$0.05	$0.30
Conectores, S_Sen1..4, S_Barrera...	Pin Headers Male 1 × 4, 1 × 3, 1 × 2, 1 × 1	Non-specific	DigiKey/Mouser	20	$0.08	$1.50
STRUCT2	PLA Parking Base & Gate Infrastructure	Polymer	Self-printed (FDM)	1	$18.00	$18.00

**Module 3: Drawbridge**						
Fuente Arduino	Arduino UNO R3 Core	Semiconductor	Adafruit/Retailers	1	$28.00	$28.00
Puente	DS3218 (20 kg-cm Metal Gear Servo)	Non-specific	SparkFun/Online	1	$18.00	$18.00
Banda	MG996R (360° Continuous Servo)	Non-specific	SparkFun/Online	1	$10.00	$10.00
Laser, LDR	KY-008 Laser Transmitter + LDR Module	Semiconductor	DigiKey/Mouser	1	$2.00	$2.00
BRG1–BRG3	XIKE 6004-2RS Double-Rubber Bearings	Metal	XIKE/Online Store	3	$2.00	$6.00
BASE1	Laser-cut MDF Platform + Grass Cover	Organic	Local Timber Workshop	1	$16.00	$16.00
REG1	LM2596 Step-Down Buck Module (8V Output)	Semiconductor	SparkFun/Online	1	$8.00	$8.00
PCB3	Altium Single-layer Custom PCB	Composite	Custom PCB Bureau	1	$2.00	$2.00
D1	1N4004 Rectifier Diode	Semiconductor	DigiKey/Mouser	1	$0.20	$0.20
Jack DC	14 × 9mm Jack DC Panel Mount	Non-specific	DigiKey/Mouser	1	$1.00	$1.00

LED1, LED2	3 mm Red LEDs Visual Signaling	Semiconductor	DigiKey/Mouser	2	$0.10	$0.20
R1, R3	330Ω 1/4 W Limiting Resistors	Semiconductor	DigiKey/Mouser	2	$0.05	$0.10
R2	1kΩ 1/4 W LDR Divider Resistor	Semiconductor	DigiKey/Mouser	1	$0.05	$0.05
S_Banda, S_Laser, S_LDR, S_Puente...	Pin Headers Male 1 × 3, 1 × 2, 1 × 1	Non-specific	DigiKey/Mouser	13	$0.08	$1.00
STRUCT3	PLA Bridge Dynamic Sections 3D Structure	Polymer	Self-printed (FDM)	1	$27.00	$27.00

**Module 4: Automated Food Dispenser**						
Arduino	Arduino UNO R3 Core	Semiconductor	Adafruit/Retailers	1	$28.00	$28.00
SERVO	DS3218 (20 kg-cm Metal Gear Servo)	Non-specific	SparkFun/Online	1	$18.00	$18.00
MODULO	DS3231 High-Precision I2C RTC Module	Semiconductor	Adafruit/Retailers	1	$4.00	$4.00
i2c_LCD	LCD 16 × 2 Display Panel + I2C Adapter Base	Semiconductor	SparkFun/Online	1	$5.00	$5.00
Bomba	5 V DC Liquid Pump + Silicon Hose	Polymer	SparkFun/Online	1	$8.00	$8.00
S_rele	5 V Single-Channel Relay Module	Semiconductor	SparkFun/Online	1	$3.00	$3.00
Sen_agua	Resistance Depth Sensor Probe	Semiconductor	DigiKey/Mouser	1	$1.00	$1.00
RES1	Hermetic Airtight Plastic Container	Polymer	Upcycled Retail	1	$5.00	$5.00
PNL1	Precision Laser-cut Transparent Acrylic Sheet	Polymer	Local Plastics Center	1	$10.00	$10.00
7805	LM7805 Positive Voltage Regulator IC	Semiconductor	DigiKey/Mouser	1	$0.80	$0.80
100nf, 470uf	100nF Ceramic + 470μF Alum. Capacitors	Semiconductor	DigiKey/Mouser	2	$0.20	$0.40
D1	General-purpose Rectifying Diode	Semiconductor	DigiKey/Mouser	1	$0.20	$0.20
Puertos (KF3)	KF3-5.08 Pitch PCB Screw Terminal Block	Polymer	DigiKey/Mouser	1	$0.50	$0.50
R1, R2	220Ω 1/4 W Pull-up Resistors	Semiconductor	DigiKey/Mouser	2	$0.05	$0.10
JP1, JP2, JP4	Angled Socket Headers WR-PHD 2.54 mm	Non-specific	DigiKey/Mouser	13	$0.12	$1.50
PCB4	Altium Single-layer Custom PCB	Composite	Custom PCB Bureau	1	$8.00	$8.00
STRUCT4	PLA Hopper Structure & Volumetric Valve	Polymer	Self-printed (FDM)	1	$30.00	$30.00

**Module 5: Home Automation (Domotics)**						
U1	Arduino UNO R3 Core	Semiconductor	Adafruit/Retailers	1	$28.00	$28.00
J16, J28 (RFID)	RFID RC522 High-Frequency Module	Semiconductor	SparkFun/Online	1	$4.00	$4.00
J9, J13 (PUERTA, SERVO)	Micro Servos SG90 (9g) Door Actuators	Non-specific	Adafruit/Online	2	$3.00	$6.00
J3 (PIR)	PIR HC-SR501 Proximity Sensor	Semiconductor	SparkFun/Online	1	$2.00	$2.00
J17 (MODULO IR)	IR Integrated Receiver + Remote	Semiconductor	SparkFun/Online	1	$3.00	$3.00
J6 (DTH11)	DHT11 Digital Atmospheric Telemetry Sensor	Semiconductor	SparkFun/Online	1	$5.00	$5.00
J8 (BUZZER)	Passive/Active Buzzer Transducer Set	Semiconductor	DigiKey/Mouser	2	$0.50	$1.00
J7 (M.DC)	5 V DC Core Motor + Propeller Assembly	Non-specific	DigiKey/Mouser	1	$2.00	$2.00
R4 (POT)	5kΩ Linear Rotary Potentiometer	Semiconductor	DigiKey/Mouser	1	$0.80	$0.80
J24 (LCD)	LCD 16 × 2 Display Panel + I2C Adapter Base	Semiconductor	SparkFun/Online	1	$5.00	$5.00

J1, J4, J5 (SW M., SWITCH, SW VIN)	Heavy-duty Toggle Switches + Shielding	Non-specific	DigiKey/Mouser	3	$3.33	$10.00
BASE1	Laser-cut Structural MDF Enclosure Board	Composite	Local Timber Workshop	1	$18.00	$18.00
U1, U2	7805 Fixed Regulator + LM317 Adjustable	Semiconductor	DigiKey/Mouser	2	$0.90	$1.80
C1–C4	Caps: .033μF (C1), 0.1μF (C2, C3, C4)	Semiconductor	DigiKey/Mouser	4	$0.12	$0.50
D1, D2	1N4007 General-purpose Rectifying Diodes	Semiconductor	DigiKey/Mouser	2	$0.20	$0.40
Q1, Q3	2N2222A NPN Switching Transistors	Semiconductor	DigiKey/Mouser	2	$0.30	$0.60
Q2	IRFZ44 N-Channel Power MOSFET	Semiconductor	DigiKey/Mouser	1	$1.00	$1.00
R1, R2, R3, R5, R6	Resistors: 1kΩ (R1, R2), 220Ω (R3), 470Ω (R5), 100kΩ (R6)	Semiconductor	DigiKey/Mouser	5	$0.08	$0.40
J2, J10, J11, J12, J15, J18	Molex 2-pin/3-pin Headers, CON18 SIP	Non-specific	DigiKey/Mouser	26	$0.10	$2.50
PCB5	Altium Single-layer Custom PCB Layout	Composite	Custom PCB Bureau	1	$12.00	$12.00
STRUCT5	PLA Architectural Walls 3D Structure	Polymer	Self-printed (FDM)	1	$50.00	$50.00

**General Infrastructure**						
PWR1	12V-5 A DC-DC Industrial Power Adapter	Non-specific	Adafruit/Online	1	$15.00	$15.00
FUSE1	Inline Fuse Box Housing + 5 A Fast Fuses	Polymer	DigiKey/Mouser	1	$5.00	$5.00

**General Assembly Hardware & Fasteners (All Stations)**						
SCRW1	M3 Carbon Steel Screws Pack (8–25 mm)	Metal	Hardware Supplier	100	$0.04	$4.00
NUT1	M3 Standard Steel Fasteners Hexagonal Nuts	Metal	Hardware Supplier	100	$0.02	$2.00

## 5 Build instructions

The physical construction and synthesis of the open-source modular experimentation kit are executed through a decoupled toolchain, ensuring that each educational station can be fabricated independently. To satisfy replication criteria while maintaining manuscript readability, step-by-step visual assembly sequences are modularly compartmentalized into five standalone User and Assembly Manuals provided in the open-access data repository. However, to provide immediate structural insight within this manuscript, individual high-resolution CAD-rendered explosion and assembly diagrams detailing the mechanical mating interfaces are integrated directly within each module’s specific assembly framework. This section outlines the standardized engineering parameters, fabrication workflows, and critical module integration steps required for full hardware replication.

### 5.1 Mechanical fabrication and slicing parameters

All structural components, brackets, and functional enclosures are optimized for additive manufacturing using Fused Deposition Modeling (FDM) technology. The structural parts were modeled parametrically to minimize post-processing overhead by avoiding internal support material across 90% of the geometries. The digital models (.STEP) must be translated to mesh profiles (.STL) and sliced under the following standardized manufacturing configurations:

• **Material Selection:** Standard Polylactic Acid (PLA) filament was utilized due to its dimensional stability and low thermal contraction coefficient during indoor handling.
• **Layer and Wall Properties:** Layer height set to 0.2 mm, with a minimum of three (3) perimeter walls to ensure mechanical structural strength against continuous student manipulation.
• **Infill Density:** A 15% infill density utilizing a gyroid pattern configuration was implemented, maximizing the strength-to-weight ratio of the chassis bases.
• **Production Dynamics and Timeframe:** The estimated FDM 3D printing execution time ranges between 8 to 12 h per module, fundamentally scaling based on the absolute physical volume and footprint of each structural chassis.
• **Subtractive Structural Components:** For high-scale structural elements requiring planar stability, such as the main chassis base of Module 3 (Drawbridge) and Module 5 (Home Automation), the vector boundaries (.DXF) are optimized for precision CNC laser cutting utilizing 3 mm and 6 mm Medium-Density Fibreboard (MDF) and clear Acrylic sheets.

### 5.2 Electronic fabrication and slicing specifications

To safeguard the system electronics from loose connections, intermittent short circuits, or contact wear during station rotations, custom single-layer Printed Circuit Boards (PCBs) were developed in Altium Designer.

1. **PCB Production:** The compressed manufacturing files (Gerber X2 and NC Drill) must be extracted and uploaded to a circuit prototyping bureau. Traces are routed with a conservative 0.4 mm minimum width and 0.5 mm clearance boundaries to allow regional chemical etching if commercial fabrication is unavailable.
2. **Component Solder Routing:** Solder joints must be executed utilizing standard lead-free tin alloy wires (SAC305 configuration) with an electronic soldering iron regulated between 320 °C and 350 °C.
3. **Visual Cross-Reference:** The high-contrast monochrome assembly PDFs provided in the repository (Artwork.pdf files) must be used as static physical templates to verify component polarity (diodes, electrolytic capacitors, and integrated circuit orientations) prior to securing the pins.

### 5.3 Assembly of specific modules

**Fig. 6:**
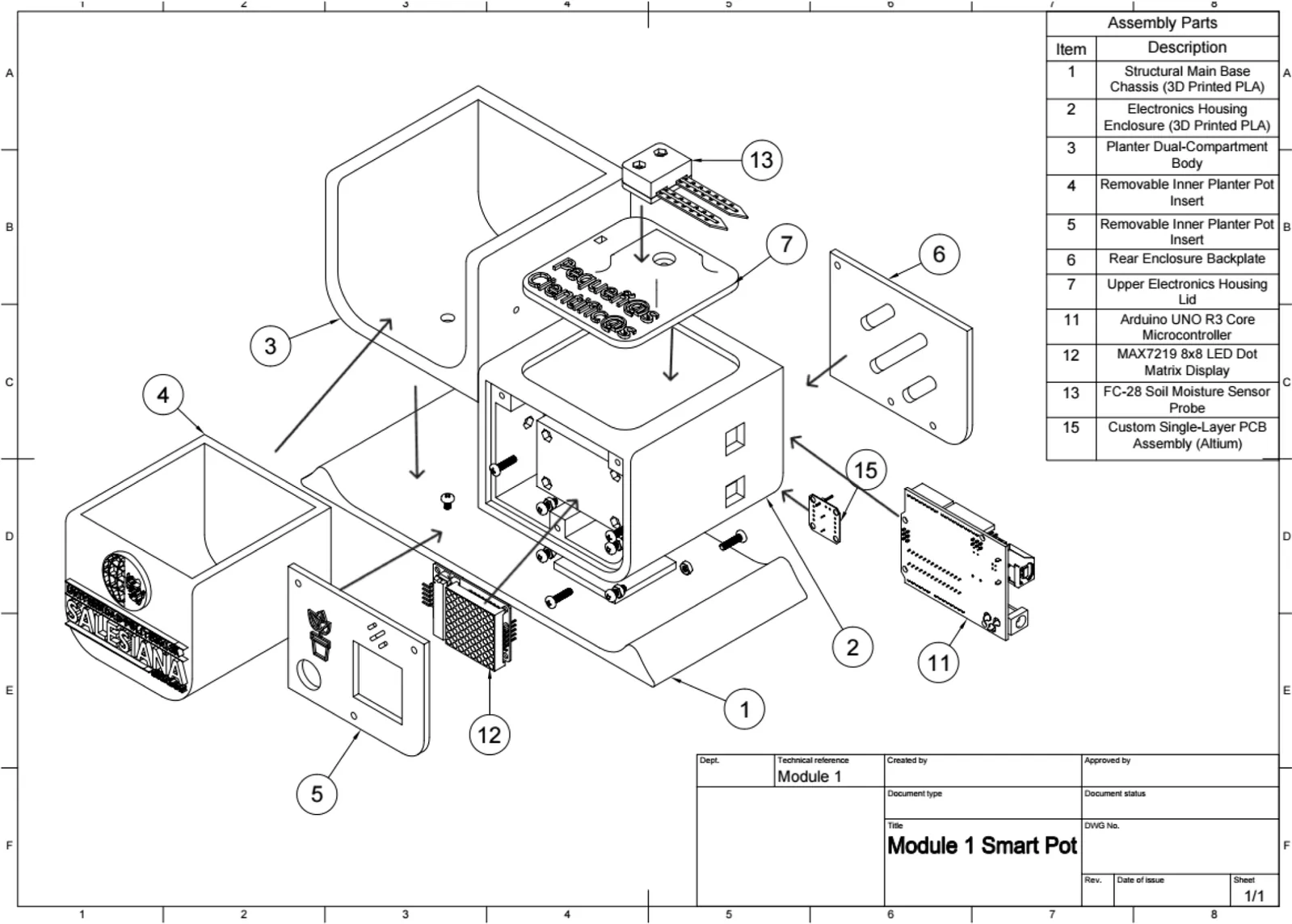
Engineering assembly drawing of Module 1 (Smart Flower Pot), featuring exploded geometric views, balloon callout tags, custom Altium PCB positioning, and the associated Assembly Parts List.

**Fig. 7:**
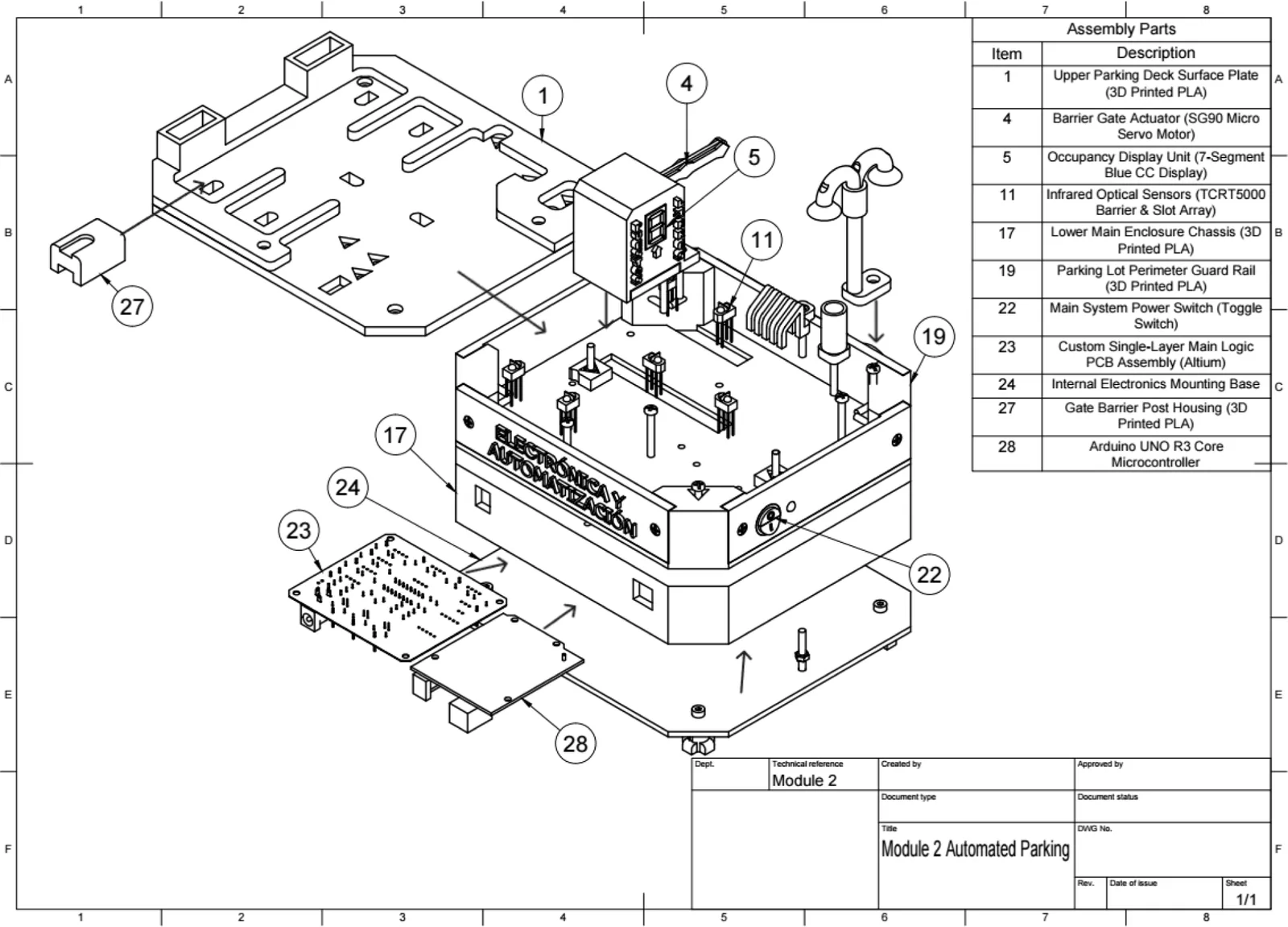
Engineering assembly drawing of Module 2 (Automated Parking System), illustrating exploded geometric views, balloon callout tags, and the associated Assembly Parts List.

**Fig. 8:**
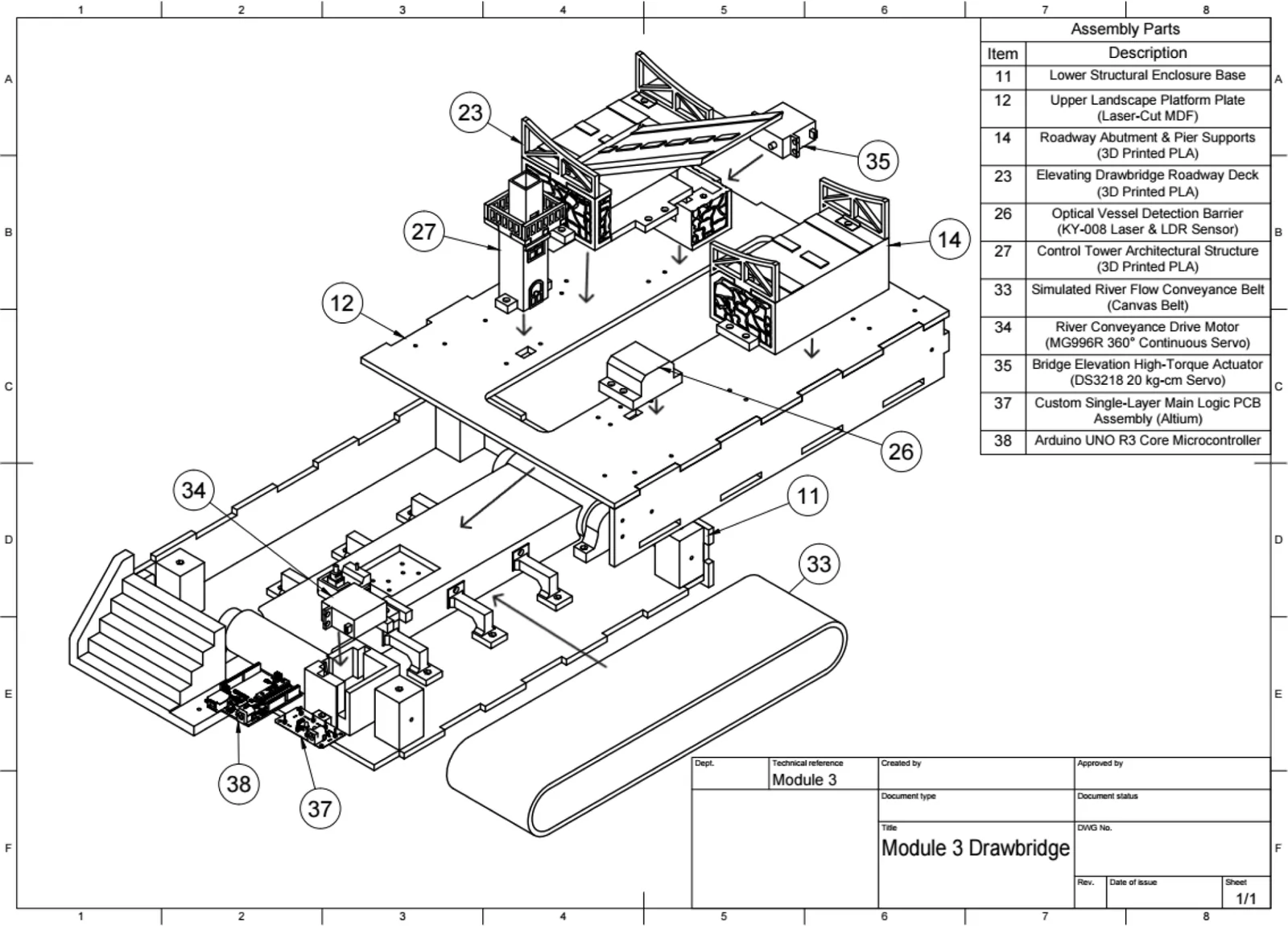
Engineering assembly drawing of Module 3 (Electromechanical Drawbridge), illustrating exploded geometric mechanics, balloon callout tags, and the associated Assembly Parts List.

**Fig. 9:**
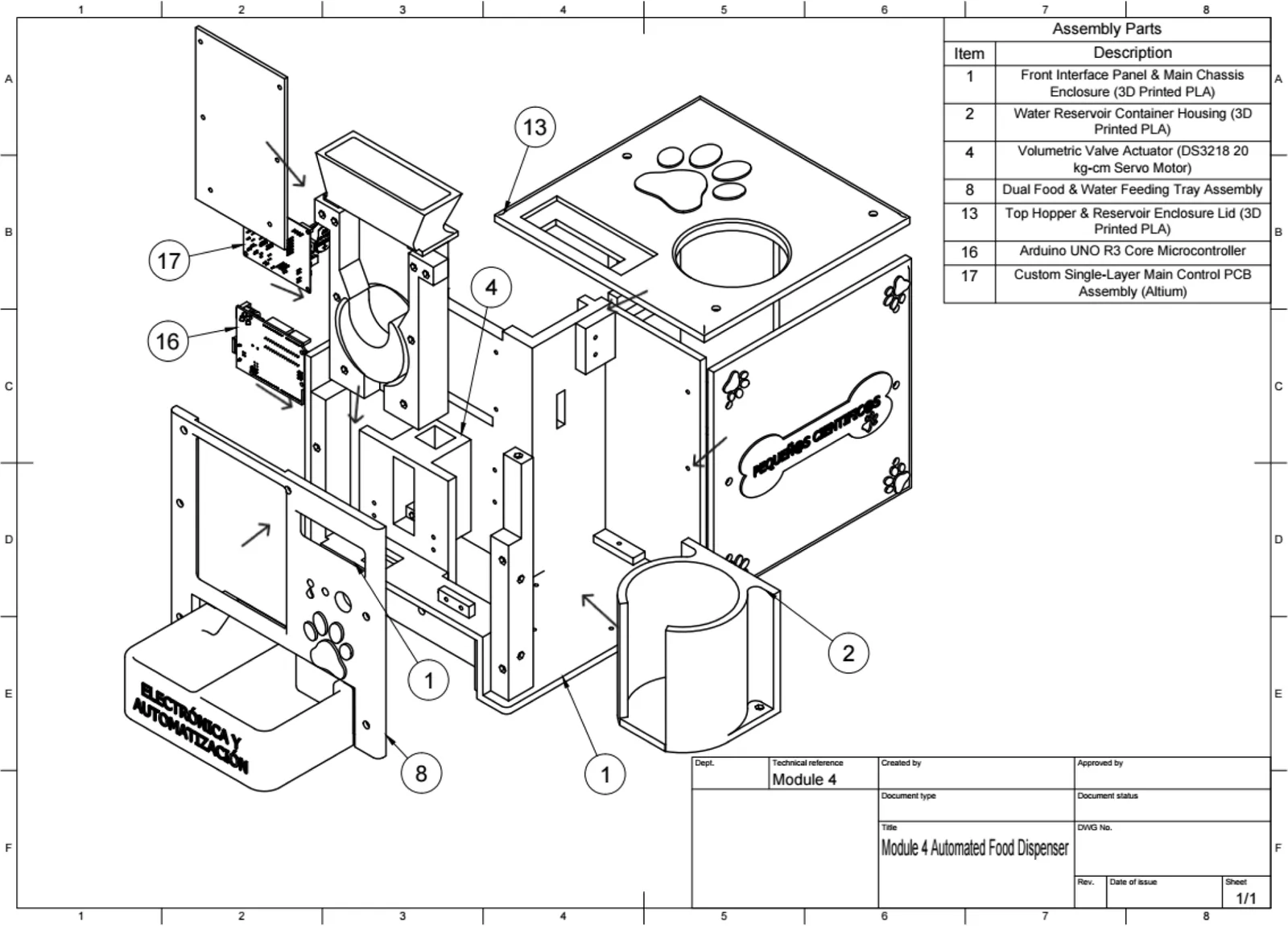
Engineering assembly drawing of Module 4 (Automated Food Dispenser), featuring exploded geometric mechanics, balloon callout tags, and the associated Assembly Parts List.

**Fig. 10:**
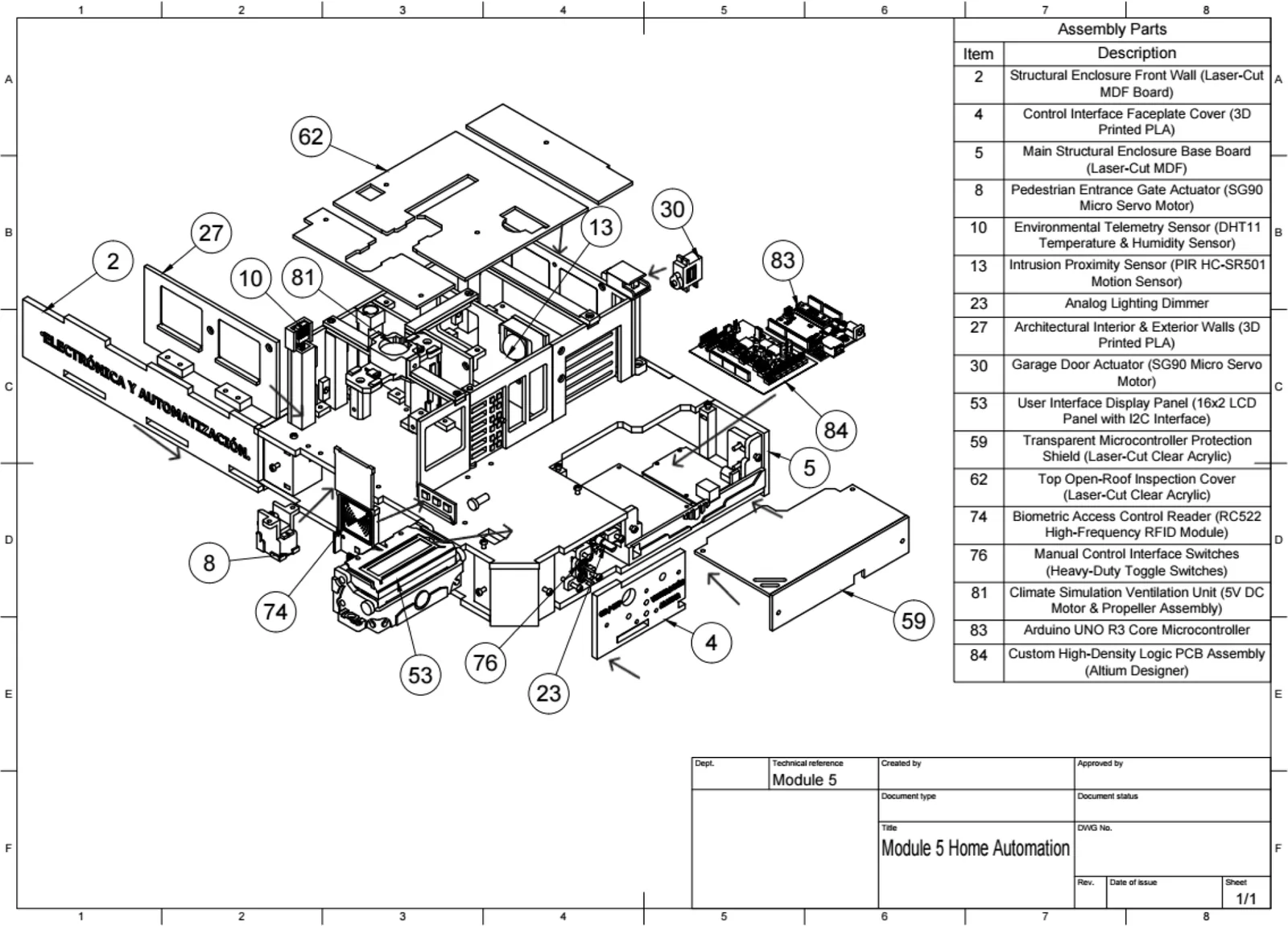
Engineering assembly drawing of Module 5 (home automation system), showcasing exploded architectural views, balloon callout tags, and the associated Assembly Parts List.

1. **Module 1 (Smart Pot):** The MAX7219 8 × 8 LED matrix is press-fitted into the dedicated front bezel of the 3D-printed electronics housing. The FC-28 capacitive moisture probe is routed through the upper isolating orifice into the separate planter base, keeping the electronic bus safely isolated from water ingress. The complete mechanical stack, fastener alignment, and structural mating zones for this environmental unit are detailed in Fig. 6.
2. **Module 2 (Automated Parking):** The TCRT5000 infrared sensor brackets are mounted at a fixed 45° angle underneath the primary platform roadway to minimize ambient light saturation. The SG90 micro-servo is linked to the physical barrier gate using standard M3 metric machine screws. Fig. 7 outlines the internal concealed routing chambers beneath the parking slots.
3. **Module 3 (Drawbridge):** The MG996R continuous rotation servo is coupled directly to the active conveyor roller axle, supported by two XIKE 6004-2RS double-rubber ball bearings to prevent belt misalignment. The high-torque DS3218 lifting actuator is mounted horizontally to the MDF structural base plate. The mechanical load distribution and bearing alignment frameworks are visualized in Fig. 8.
4. **Module 4 (Automated Food Dispenser):** The internal rotary gate valve must be mechanically aligned with the DS3218 servo horn locked at its absolute 30° indexing point (standby closed state) before tightening the fasteners. The modified airtight fluid container must be sealed to ensure a leak-proof path for the 5 mm suction hose. Fig. 9 provides the exact orientation of the hopper valve.
5. **Module 5 (Home Automation System):** Black 3D-printed conduits are fitted onto the main MDF board to route the dense 11-peripheral wiring network beneath the residential layout. The RC522 RFID reader is placed inside its dedicated front patio compartment, while the PIR and IR modules are elevated to avoid crosstalk. The absolute spatial layout and component density are mapped in Fig. 10.

## 6 Operation instructions

### 6.1 Software deployment environment and library synchronization

The open-source control firmware for all modules was developed using the C++ framework within the **Arduino IDE (version 2.3.2)**. To guarantee compilation repeatability and eliminate driver mismatches, the specific library versions included in the repository’s Shared_Libraries folder must be imported manually into the environment via *Sketch -*> *Include Library -*> *Add .ZIP Library*.

• **Actuator Control Bus:** Servo.h (Native release v1.2.1) managing positional PWM curves.
• **Matrix Display Controller:** LedControl.h (v1.0.6) utilized for low-level register manipulation of the MAX7219 8 × 8 matrix sub-system.
• **Infrared Remote Receiver:** IRremote.h (v4.4.0) executing logic capture for multi-protocol handheld IR telemetry tokens.
• **Prerequisite Core Abstraction:** Adafruit_Sensor.h (v1.1.5) establishing unified sensor baseline classes.
• **HMI Interfacing:** LiquidCrystal_I2C.h (v1.1.2) hardcoded to register hex address 0x3F.
• **Chronological Tracking:** RTClib.h (Adafruit driver v2.1.4) executing I2C time registration registers.
• **Security Registers:** MFRC522.h (v1.4.12) managing high-frequency SPI transponder tokens packaged inside the local MFRC522.zip dependency.
• **Climatic Abstraction:** DHT.h (v1.4.7) parsing environmental telemetry fields.

### 6.2 Functional operational logic per module

#### 6.2.1 Module 1: Smart Pot execution

The system executes an ongoing analog polling routine of the soil moisture levels through the FC-28 sensor every 2000 ms. The raw internal analog threshold is set to an arbitrary value of 400 points within the code:

• If Moisture≥400, the microcontroller parses a predefined binary matrix array to project a “happy face” icon onto the MAX7219 matrix display.
• If Moisture<400, the system refreshes the screen data to project a “sad face” warning icon, notifying the user to initiate irrigation.

#### 6.2.2 Module 2: Automated parking control

The system treats slot management through an ongoing absolute state-machine loop. Four embedded slot IR sensors scan space availability. The active vehicle count is calculated dynamically (Available=Total_Slots−Occupied). The Arduino outputs a 4-bit binary code representing this digital value directly to the 74LS48 hardware BCD-to-7-segment decoder, updating the display metrics without computational lag. When the entrance IR sensor detects an incoming chassis, the gate servo barrier sweeps to 130° for a 5-second interval before returning to the 165° standby state.

#### 6.2.3 Module 3: Drawbridge actuation loop

Upon toggling the main hardware switch, the continuous conveyor servo is driven at a fixed velocity array index of 100 to rotate the river canvas. The system samples telemetry data from the LDR analog optical barrier. When a structural vessel blocks the KY-008 laser beam, the analog baseline voltage rises above the 400-point safety threshold. The microcontroller halts the conveyor belt, activates the bridge LED warning light, and initiates a smooth incremental loop sweeping the DS3218 servo from 48° to 90° over 1260 ms. The vertical state is held for 10 s before executing a decrementing angle loop to safely lower the deck.

#### 6.2.4 Module 4: Automated food dispenser routine

The control logic relies on the continuous reading of the DS3231 RTC registers. The firmware evaluates time arrays against three daily scheduled benchmarks: Breakfast (08:00), Lunch (15:57), and Dinner (09:23). When a match is validated, an internal boolean flag locks execution to prevent double-triggering, and the DS3218 servo executes a controlled sweep from 30° to 170°, holding the valve open for 3000 ms to drop a metered volumetric food portion. Concurrently, the liquid depth sensor evaluates water levels; if values drop below 100 points, the 5 V relay isolates and engages the liquid pump for 5 s.

#### 6.2.5 Module 5: Home automation hub interfacing

The capstone module executes parallel sub-routines to manage domestic devices:

• **RFID/IR Security:** The RC522 scanner monitors for proximity tokens; if the read UID matches the internal array whitelist (3d38c052), the entrance servo unlatches by sweeping to 180°. Alternatively, receiving the 0xFFA25D hex code via the IR receiver actuates the garage gate.
• **Climate Automation:** The DHT11 sensor tracks local atmospheric metrics; if the temperature registers ≥10°C, the 5 V DC fan motor is driven high.
• **Intrusion Detection:** If the PIR sensor flags motion while the security switch is armed, a loop toggles the ALARM_PIN buzzer high/low in 500 ms safety intervals.

### 6.3 Safety considerations and operational constraints

Due to the implementation of high-torque servo motors (such as the DS3218) in the mechanical actuators, peak current draw may temporarily exceed 2.0 A exclusively under severe mechanical stall or external jamming conditions. To prevent thermal overhead, voltage sags, or power supply protection trip, the following operational constraints must be strictly followed:

1. A maximum structural and operational payload limit of 500 grams is enforced on the Drawbridge deck module. Users must strictly avoid applying manual external opposition or holding down the moving structures during actuation loops.
2. Firmware routines incorporate absolute physical limits (e.g., restricted angular sweeps between 30° and 170° for the Food Dispenser) to avoid mechanical binding against the 3D-printed chassis frameworks.
3. Users must isolate the microcontroller power from the actuator power rails during multi-peripheral continuous testing to shield the logic gates from inductive kickback.

### 6.4 User guides

To facilitate the adoption of this hardware in schools, a set of illustrated “User Guides” was developed (see Mendeley Data Repository / Table 2). These guides translate the technical operation described above into student-friendly flowcharts and step-by-step diagrams, bridging the gap between engineering specifications and classroom usability.

## 7 Validation and characterization

To verify the utility, robustness, and educational appeal of the hardware, a field validation was conducted. As noted by standard educational hardware assessments, evaluating deep conceptual learning during short interventions is challenging; therefore, this validation explicitly focused on measuring the system’s functional reliability and the students’ level of engagement and motivation.

### 7.1 Experimental setup and participant demographics

The physical hardware ecosystem was tested during a continuous 6-hour practical workshop infrastructure. To optimize hardware allocation constraints and foster collaborative learning dynamics, a station-based laboratory rotation methodology was implemented. The experimental classroom space was divided into five standalone workstations, each permanently deploying one of the five specific modules.

A total of 40 secondary school students participated in the field validation track. The cohort was divided into 5 experimental groups of exactly 8 students per active module workstation. Groups rotated through the internal technical stations systematically, ensuring that all participants obtained hands-on execution and hardware manipulation experience with each module. The 6-hour baseline session was structured into an initial theoretical framework introduction (1 h), the specialized mechanical and electronic assembly phase (3 h), and a final functional validation, deployment, and firmware coding phase (2 h). The students were selected from a standard academic secondary track without prior specialized technical training in electronics or microcontrollers. Table 4 summarizes the participant demographics and global workshop setup parameters; the age bracket metrics reflect a rigorous descriptive entry synchronization from the active school baseline database without altering the fixed sample size (N=40).

**Table 4:** Participant Demographics and Setup Summary (N=40).

Parameter	Value
Total Participants	40
Gender Distribution	16 Female (40%), 24 Male (60%)
Age Range	15 – 18 years
Mean Age	16.2 years (SD = 0.64)
Grade Level	10th to 12th Grade (Secondary Education)
Methodology	Station-based collaborative rotation
Team Size	8 students per module workstation
Instrument Validity	Expert judgment & behavioral observation

### 7.2 Functional reliability assessment

The physical robustness was evaluated through direct observation. The 3D-printed PLA structural components were subjected to multiple assembly cycles. No mechanical failures or cracks were recorded. Furthermore, the use of custom PCBs and DuPont connectors minimized common failure modes such as loose wiring or short circuits. During the 6-hour session, 100% of the modules remained operationally functional under continuous student handling.

### 7.3 User engagement and interest assessment

To quantify student interaction properties, a comprehensive post-workshop descriptive evaluation was administered. For the purpose of this study, *student engagement* is formally defined as a multi-dimensional construct modeling student scientific curiosity, active interaction, and positive motivation toward automation engineering concepts. Consequently, a post-workshop survey instrument was deployed using a standardized 5-point Likert scale, where 1 represents *Strongly Disagree* and 5 represents *Strongly Agree*. The evaluation strictly mapped four core operational constructs: Ease of Use, Active Participation, Curiosity, and STEM Interest.

Table 5 displays the detailed descriptive evaluation statistics. The metrics recorded in the primary dataset correspond directly to the absolute, non-transformed Likert scale responses reported by the participants. To facilitate comparative evaluation alongside regional literature benchmarks, these absolute scores were structurally normalized into percentage-based interest metrics utilizing a standard linear metric scaling function, mathematically expressed as follows:

(1) $$\text{Normalized Interest (\%)}=\left(\frac{\text{Mean Likert Score}}{5}\right)\times 100\text{\%}$$

The empirical results confirm high levels of baseline engagement across all experimental stations.

**Fig. 11:**
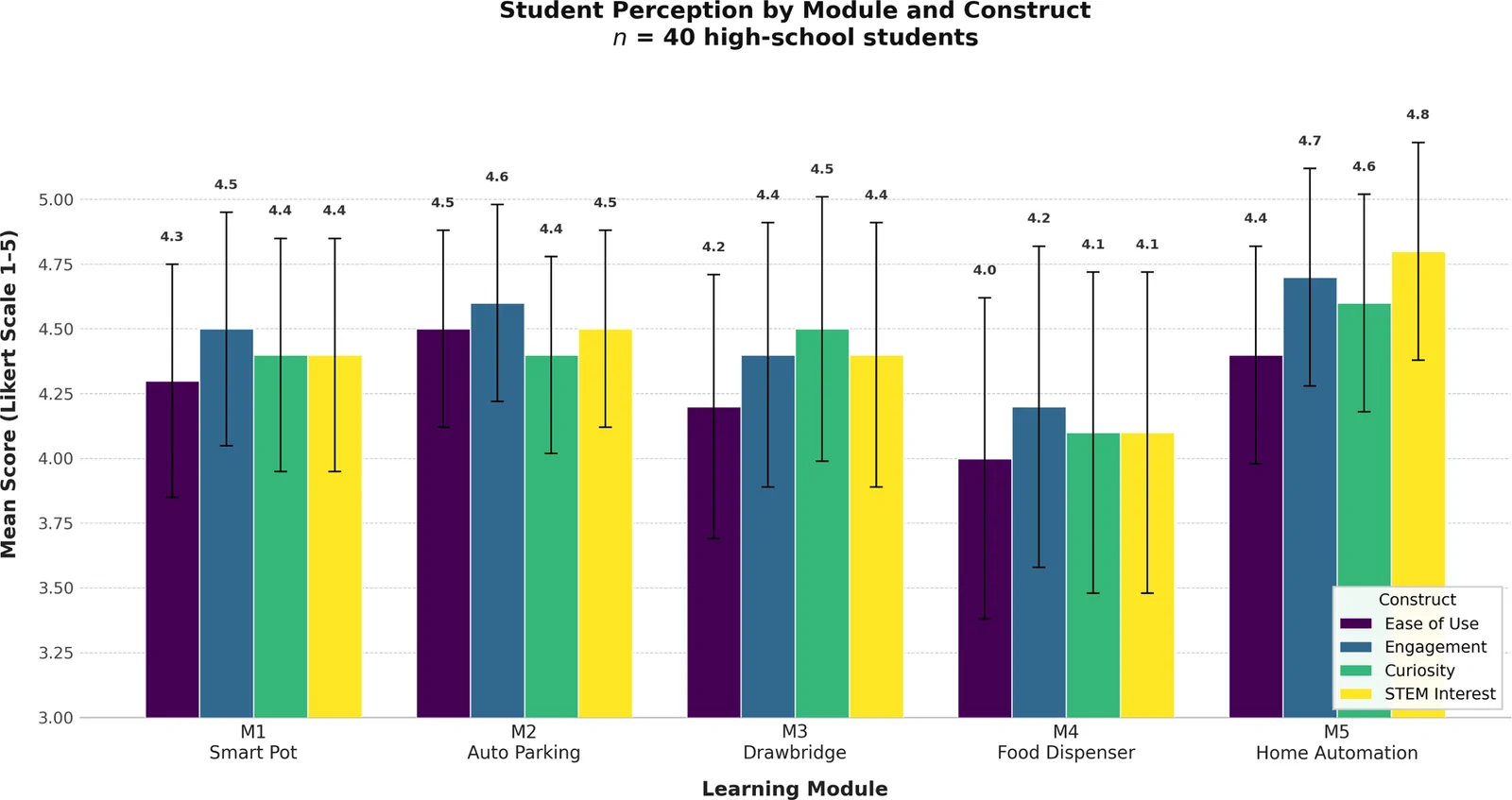
Detailed motivation scores by construct and module. Error bars represent standard deviation (±SD). High scores across all constructs confirm the kit’s success in fostering STEM interest.

**Fig. 12:**
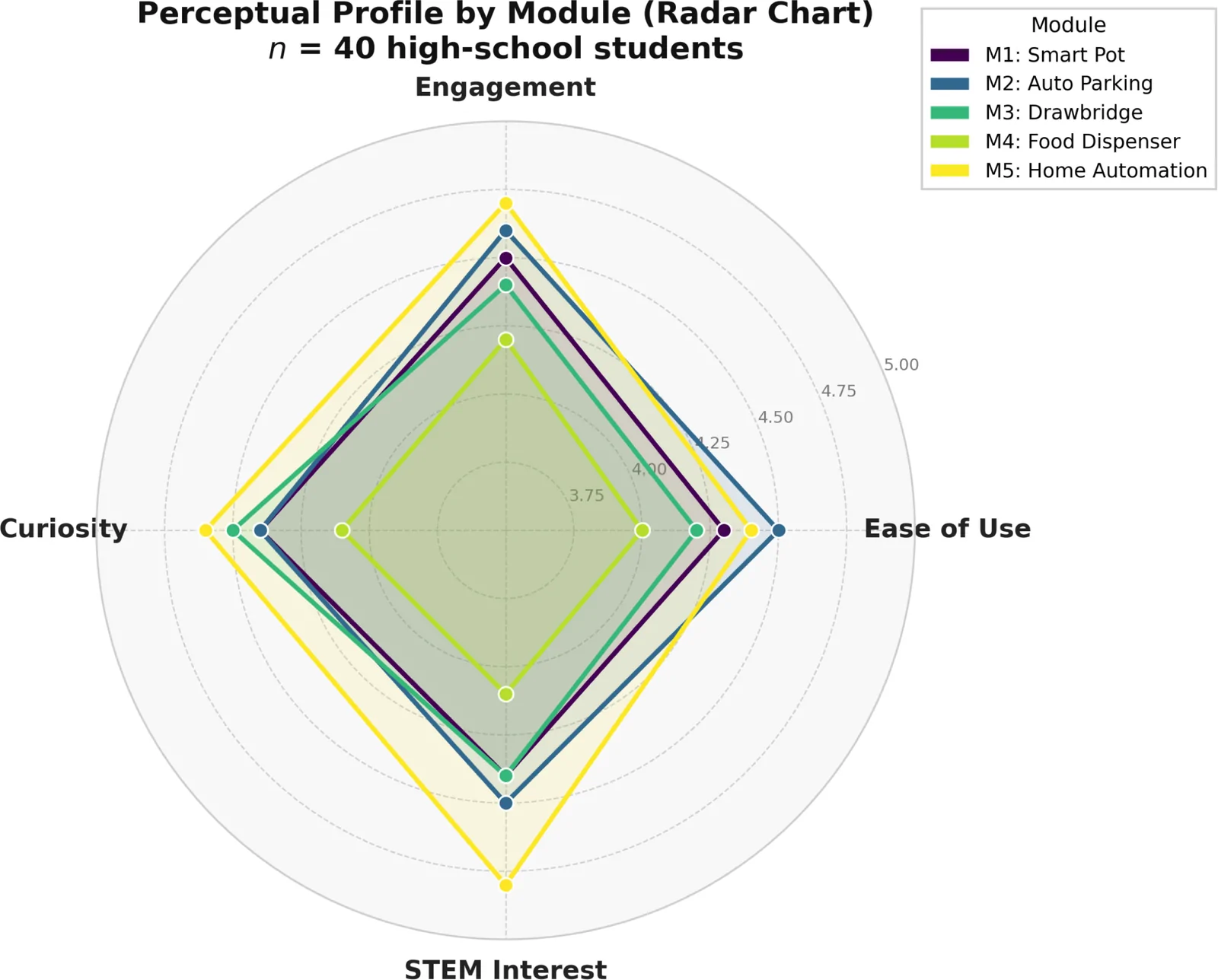
Radar chart showing the balance between constructs for each module. The overlap suggests a consistent instructional design across the entire experimentation kit.

The quantitative scores are further visualized via the structural construct distribution in Fig. 11 and the perceptual profile mapped in Fig. 12. A deep cross-examination of these graphical trends reveals that modules incorporating immediate, dynamic telemetry or real-time human-machine interface (HMI) feedback — specifically Module 5 (Home Automation) with a mean score of 4.51 ± 0.42 and Module 2 (Automated Parking) with a mean score of 4.48 ± 0.38 — achieved the most prominent engagement metrics. This behavior suggests that visual or acoustic physical actions (such as LCD readouts, automated barrier movements, and alarm toggles) significantly boost student curiosity and active interaction.

**Table 5:** User Engagement and Interest Results by Module (N=40).

Module name	Interest (%)	Likert Score (1–5)	Standard deviation (±SD)
M1: Smart Flower Pot	88.1%	4.41	0.45
M2: Automated Parking	89.5%	4.48	0.38
M3: Drawbridge	87.6%	4.38	0.51
M4: Food Dispenser	82.0%	4.10	0.62
M5: Home Automation	90.2%	4.51	0.42

Conversely, Module 4 (Automated Food Dispenser) exhibited the lowest relative mean score (4.10 ± 0.62), while maintaining a high curiosity sub-construct trajectory. This specific deviation indicates that while time-scheduled mechanisms and fluid-handling systems introduce a higher perceived mechanical assembly challenge for secondary students, they successfully preserve the underlying scientific curiosity vector. The narrow distribution of the standard deviation (±SD) bars highlights the statistical consistency of the positive user perception across the entire experimental deployment.

The survey instrument was validated through expert judgment by senior faculty members in the Electronics Department to ensure content validity. Reliability was consistency-checked through direct observation during the station rotation, ensuring that students’ self-reported motivation matched their behavioral engagement levels in the laboratory. The negatively skewed distribution of scores confirms that the majority of students perceived the hardware as highly engaging.

### 7.4 Limitations and future work

While the validation confirmed high engagement, certain limitations were identified. The 6-hour format restricts the depth of programming instruction. Additionally, while PLA is robust for indoor use, its thermal stability is limited for long-term outdoor sensing (e.g., the Smart Pot). Future work will focus on integrating ESP32 modules for cloud-based data logging and conducting long-term longitudinal studies to assess knowledge retention.

## 8 Conclusions

The open-source 5-module modular experimentation kit successfully addressed the accessibility gaps in STEM instruction within developing socioeconomic frameworks. The physical infrastructure demonstrated 100% operational reliability under continuous, station-based collaborative rotation workshop operations under real student handling. As evidenced in the descriptive results (Table 5), modules incorporating immediate dynamic or visual telemetry feedback (such as the Home Automation and Automated Parking layouts) achieved the highest motivation indices among secondary school students.

The modular architecture successfully stimulated student scientific curiosity, demonstrating high levels of engagement and positive motivation towards automation engineering concepts without overclaiming long-term cognitive skill acquisition during a brief workshop exercise. Future work will focus on scaling the peripheral architecture to support cloud-based Internet of Things (IoT) data logging under the same low-cost open-source manufacturing principles.

## CRediT authorship contribution statement

**Luis Calle-Arévalo:** Conceptualization, Methodology, Software. **Juan Francisco Gonzales:** Data curation, Writing- Original draft preparation. **John Alonso Ortega Naula:** Visualization, Investigation, Software. **Diego Gabriel Zamora Lituma:** Visualization, Investigation, Software.

## Ethics statements

Ethical review and approval for this study were waived due to the use of anonymized secondary data, in accordance with Ecuador’s Organic Law on Personal Data Protection (Art. 7, paragraph 4) and the Universidad Politécnica Salesiana’s Policy on the Processing, Protection, and Use of Personal Data and Classification of Information (Resolution No. 128-104-2023-04-19).

## Funding

This research was funded by the 10.13039/100016969Universidad Politécnica Salesiana . Resolution No. 258-017-2024-07-03.

## Declaration of competing interest

The authors declare that they have no known competing financial interests or personal relationships that could have appeared to influence the work reported in this paper.
